# Comparison of the analgesic effects of ultrasound-guided erector spinae plane block and quadratus lumborum block: a systematic review and meta-analysis

**DOI:** 10.3389/fphar.2025.1640135

**Published:** 2025-08-01

**Authors:** Lin Wu, Weiyi Zhang, Yiyong Wei, Donghang Zhang

**Affiliations:** ^1^ Department of Anesthesiology, West China Hospital, Sichuan University, Chengdu, China; ^2^ Laboratory of Anesthesia and Critical Care Medicine, National-Local Joint Engineering Research Centre of Translational Medicine of Anesthesiology, West China Hospital, Sichuan University, Chengdu, China; ^3^ Department of Anesthesiology, Longgang Maternity and Child Institute of Shantou University Medical College (Longgang District Maternity and Child Healthcare Hospital of Shenzhen City), Shenzhen, China

**Keywords:** analgesia, erector spinae plane block, meta-analysis, postoperative pain, quadratus lumborum block, systematic review

## Abstract

**Background:**

Erector spinae plane block (ESPB) and quadratus lumborum block (QLB) are commonly used for perioperative analgesia in various surgeries. An increasing number of randomized controlled trials (RCTs) have compared the analgesic effect and safety of ESPB with those of QLB, but the conclusions are controversial. This study was designed to identify whether ultrasound-guided ESPB was better than the QLB for postoperative analgesia.

**Methods:**

To identify RCTs comparing ESPB with QLB for postoperative analgesia, we searched PubMed, Embase, the Cochrane Library, and Web of Science. The primary outcome was postoperative analgesic consumption over 24 h. The secondary outcomes included the time to the first analgesic request, postoperative resting pain scores, block performance time, postoperative rescue analgesia rate, incidence of complications, and postoperative satisfaction. RevMan 5.4 software was used in the analysis. Subgroup analysis and sensitivity analysis were performed to explore the source of heterogeneity and test the reliability of the pooled results. The quality of evidence was systematically assessed via the GRADE evaluation.

**Results:**

Twenty-seven studies involving 1942 patients were included. Compared with QLB, ESPB consumed fewer 24-h postoperative analgesics (WMD, −4.03; 95% CI, −6.25 to −1.82; *P* = 0.0004; moderate quality of evidence), spent less time performing blocks (WMD, −1.55; 95% CI, −2.68 to −0.41; *P* = 0.008; moderate quality of evidence), and had a lower incidence of postoperative nausea and vomiting (RR, 0.72; 95% CI, 0.58 to 0.91; *P* = 0.006; high quality of evidence). ESPB and QLB did not significantly differ in terms of time to the first analgesic request (WMD, −0.12; 95% CI, −0.47 to 0.22; *P* = 0.48; moderate quality of evidence) or postoperative resting pain scores at 6 h, 12 h, and 24 h (6 h: SMD, 0.08; 95% CI, −0.27 to 0.24; *P* = 0.66; moderate quality of evidence; 12 h: SMD, 0.13; 95% CI, −0.28 to 0.55; *P* = 0.53; moderate quality of evidence; 24 h: SMD, −0.02; 95% CI, −0.22 to 0.18; *P* = 0.87; moderate quality of evidence).

**Conclusion:**

Moderate-to high-quality evidence indicates that ESPB is superior to QLB for postoperative analgesia because of less postoperative analgesic consumption, faster block performance and a lower incidence of postoperative nausea and vomiting.

**Systematic review registration:**

https://www.crd.york.ac.uk/PROSPERO/view/CRD42024607988.

## 1 Introduction

Regional blocks play crucial roles in modern anesthesia and pain management (Fallon et al., 2024; Qin et al., 2024; Chen et al., 2021). Regional blocks provide satisfactory surgical conditions and precise anesthetic effects while extending the duration of postoperative analgesia (Kirksey et al., 2015; Joshi et al., 2016; Hade et al., 2021). Additionally, regional blocks can reduce the use of general anesthetics and opioids, avoiding the related side effects and complications associated with general anesthesia (Kirksey et al., 2015; Joshi et al., 2016; O'Flaherty et al., 2018; Zhong et al., 2024). Under contemporary anesthesia, the use of regional blocks for perioperative analgesia not only enhances the safety and efficacy of surgery but also promotes postoperative recovery and improves patients’ quality of life (Joshi et al., 2016; Héroux et al., 2023; Jogie and Jogie, 2023; Niyonkuru et al., 2024). Therefore, regional blocks have been recommended as essential components for multimodal analgesia and the practice of enhanced recovery after surgery (ERAS) (Joshi et al., 2016; Héroux et al., 2023; Jogie and Jogie, 2023).

Erector spinae plane block (ESPB) is widely used for postoperative analgesia for various surgeries, such as thoracic, abdominal, and hip surgeries, in which local anesthetics are injected into the fascial plane between the vertebral transverse processes and the erector spinae muscle (Tsui et al., 2019; Capuano et al., 2024; Coppens et al., 2023). The quadratus lumborum block (QLB) is another common type of nerve block that can be achieved by injecting local anesthetics into the fascial space near the quadratus lumborum muscle to provide perioperative analgesia (Baran et al., 2024; George et al., 2024). ESPB and QLB are both noted for their high safety, ease of performance under ultrasound guidance, and wide diffusion range (Capuano et al., 2024; Akerman et al., 2018; Ueshima et al., 2017; Kwon et al., 2020). In recent years, an increasing number of randomized controlled trials (RCTs) have compared the analgesic effects and safety of ESPB with those of QLB for postoperative pain in several types of surgeries, but the results have not been consistent (Mahmoud Fakhry et al., 2024; Hassanein et al., 2023; Barthwal et al., 2023; Taman et al., 2022; Mohasseb et al., 2021; Abd Ellatif and Abdelnaby, 2021). For the time to first analgesic request, one study (Barthwal et al., 2023) demonstrated that the ESPB group had a shorter time than the QLB group, whereas another study (Abdelaziz et al., 2024) reported the opposite outcome in adult patients undergoing hysterectomy. For postoperative analgesic consumption over 24 h, one study reported by Zhen et al. (Zhang et al., 2024) suggested that the QLB group required more analgesic consumption than the ESPB group. However, two other studies (Abd Ellatif and Abdelnaby, 2021; Onay et al., 2023) reported no difference in consumption between the two groups. Therefore, it is meaningful to conduct a systematic review and meta-analysis to determine whether ESPB was superior to QLB in postoperative analgesia, which will provide evidence for clinical choice of preferred type of regional block.

This study provides evidence-based decision-making for clinicians by quantitatively comparing the postoperative analgesic efficacy of two regional block techniques. It optimizes perioperative multimodal analgesia protocols through evidence-based medical evidence and enhances both patient recovery and overall healthcare quality.

## 2 Materials and methods

The study protocol was registered in the Prospective Register of Systematic Reviews (ID: CRD42024607988), and the study was conducted in accordance with the Preferred Reporting Items for Systematic Reviews and Meta-Analyses (PRISMA) guidelines (Moher et al., 2010). Ethical approval is not applicable.

### 2.1 Search strategies

Four databases, including PubMed, Embase, the Cochrane Library, and Web of Science, were searched from their inception to 16 October 2024. MeSH term ‘randomized controlled trial’ and free terms ‘erector spinae plane block’ and ‘quadratus lumborum block’ were used. The detailed search strategy is shown in Supplementary Material S1.

### 2.2 Study selection

Two authors (L.W. and D.Z.) separately performed study selection. All retrieved studies via search strategy were imported into EndNote V.20. Software (Clarivate Analytics, United States, 2021). After automatic and manual deduplication, the titles and abstracts of the included studies were reviewed for eligibility. Then, the full texts of remaining studies were reviewed for final inclusion. The reason for exclusion was recorded. Any discrepancies were addressed through consultation with a third author (W.Z.).

### 2.3 Inclusion and exclusion criteria

Studies were considered eligible for inclusion if (Fallon et al., 2024) they were RCTs published in English (Qin et al., 2024); they compared QLB (as the control group) with ESPB for analgesic effects; and (Chen et al., 2021) they reported any consensus-based outcomes, whether primary or secondary: postoperative analgesic consumption over 24 h (mg), time to the first analgesic request (hours), postoperative resting pain scores at 6 h, 12 h and 24 h, block performance time, postoperative rescue analgesia rate, incidence of postoperative nausea and vomiting, incidence of postoperative hypotension, and postoperative satisfaction. Satisfaction was defined as patients self-rating on a 1-5 numerical scale (1 = very dissatisfied, 5 = very satisfied). The satisfaction rate was defined as the percentage of patients who self-reported “satisfied” versus “dissatisfied”. Studies that did not meet the inclusion criteria were excluded.

### 2.4 Data extraction

Two independent researchers (L.W. and D.Z.) utilized a standardized extraction form, previously agreed upon, to gather data from eligible RCTs. The form encompassed key elements such as the title, publication year and country, first author, patient characteristics, sample size, type of surgery, interventions and control groups, and outcomes.

### 2.5 Risk of bias assessment

The assessment of bias risk in the included studies was conducted via the Cochrane Handbook-recommended Risk of Bias Assessment Tool ROB2 (revised in 2019) for RCTs. This tool evaluates five key domains of bias: the randomization process, deviations from intended interventions, missing outcome data, outcome measurement, and selection of reported results. Each domain was categorized as having a low, some concerns, or high risk of bias. Two authors independently conducted the bias risk assessments, and any discrepancies were resolved through consultation with a third author (W.Z.). The details of the risk of bias assessment are summarized in Supplementary Material S2.

### 2.6 GRADE

The GRADE evaluation system (Guyatt et al., 2008) was used to grade the quality of the combined evidence for the main outcomes, which emphasized five specific aspects (1): risk of bias (2), inconsistency (3), indirectivity (4), inaccuracy, and (5) publication bias. The GRADE evidence ranged in quality from high to very low. The details of the GRADE assessment are summarized in Table 1.

**TABLE 1:** GRADE summary.

Study outcome	Participants (studies)	Absolute effect	Quality of the evidence
Postoperative 24-h analgesic consumption (mg)	1408 (20 studies)	WMD -4.03 (−6.25 to −1.82)	⊕⊕⊕⊝ moderate^a^
Time to the first analgesic request (h)	1126 (16 studies)	WMD -0.12 (−0.47 to 0.22)	⊕⊕⊕⊝ moderate^a^
Postoperative 6 h resting pain scores	1276 (18 studies)	SMD 0.08 (−0.27–0.42)	⊕⊕⊕⊝ moderate^a^
Postoperative 12 h resting pain scores	1296 (18 studies)	SMD 0.13 (−0.28–0.55)	⊕⊕⊕⊝ moderate^a^
Postoperative 24 h resting pain scores	1461 (20 studies)	SMD -0.02 (−0.22 to 0.18)	⊕⊕⊕⊝ moderate^a^
Block performance time (min)	750 (10 studies)	WMD -1.55 (−2.68 to −0.41)	⊕⊕⊕⊝ moderate^a^
Postoperative rescue analgesia rate (%)	599 (8 studies)	RR 0.9 (0.72–1.14)	⊕⊕⊕⊕ high
Incidence of PONV^b^ (%)	1078 (16 studies)	RR 0.72 (0.58–0.91)	⊕⊕⊕⊕ high
Incidence of POH^c^ (%)	288 (4 studies)	RR 0.63 (0.32–1.25)	⊕⊕⊕⊝ moderate^d^
Postoperative satisfaction rate (%)	478 (6 studies)	RR 0.98 (0.86–1.10)	⊕⊕⊕⊝ moderate^a^
Postoperative satisfaction	432 (5 studies)	SMD -0.11 (−0.30 to 0.08)	⊕⊕⊕⊝ moderate^d^

^a^ Large I^2^-value.

^b^ PONV: postoperative nausea and vomiting.

^c^ POH: postoperative hypotension.

^d^ Small sample size.

### 2.7 Statistical analyses

RevMan V.5.4. software (The Nordic Cochrane Centre, The Cochrane Collaboration, 2014) and SPSSAU online software (Beijing Qingsi Technology Co., Ltd., China, 2016) were used for data analysis. Continuous variables are presented as the mean ± standard deviation (SD). Data reported as median and range were converted to mean ± SD via the methods described by Wan et al. (2014) and Hozo et al. (2005). The weighted mean difference (WMD) was selected to represent the effect size when the continuous variables were measured in the same way. Otherwise, the standardized mean difference (SMD) was selected. Dichotomous data were presented as the number of events, and the risk ratio (RR) was selected as the effect size. Given that postoperative analgesic consumption involves multiple opioids and routes of administration, we standardized the data by converting them to oral morphine equivalents (OMEs) before comparison (Nielsen et al., 2016). The heterogeneity of the included studies was analyzed via the chi-square test and evaluated with I^2^ statistics. A random-effects model was used due to the high heterogeneity (I^2^ > 50%). Otherwise, the fixed effects model was adopted. A sensitivity analysis was performed via the “one-by-one exclusion method” to verify the robustness of all conclusions. Subgroup analysis was conducted to explore the source of heterogeneity and to analyze the correlation between subgroup factors and conclusions. Outcomes including ≥10 studies were analyzed for publication bias via funnel plots and Begg tests. *P* < 0.05 was considered statistically significant.

## 3 Results

### 3.1 Screening process for study inclusion

**FIGURE 1:**
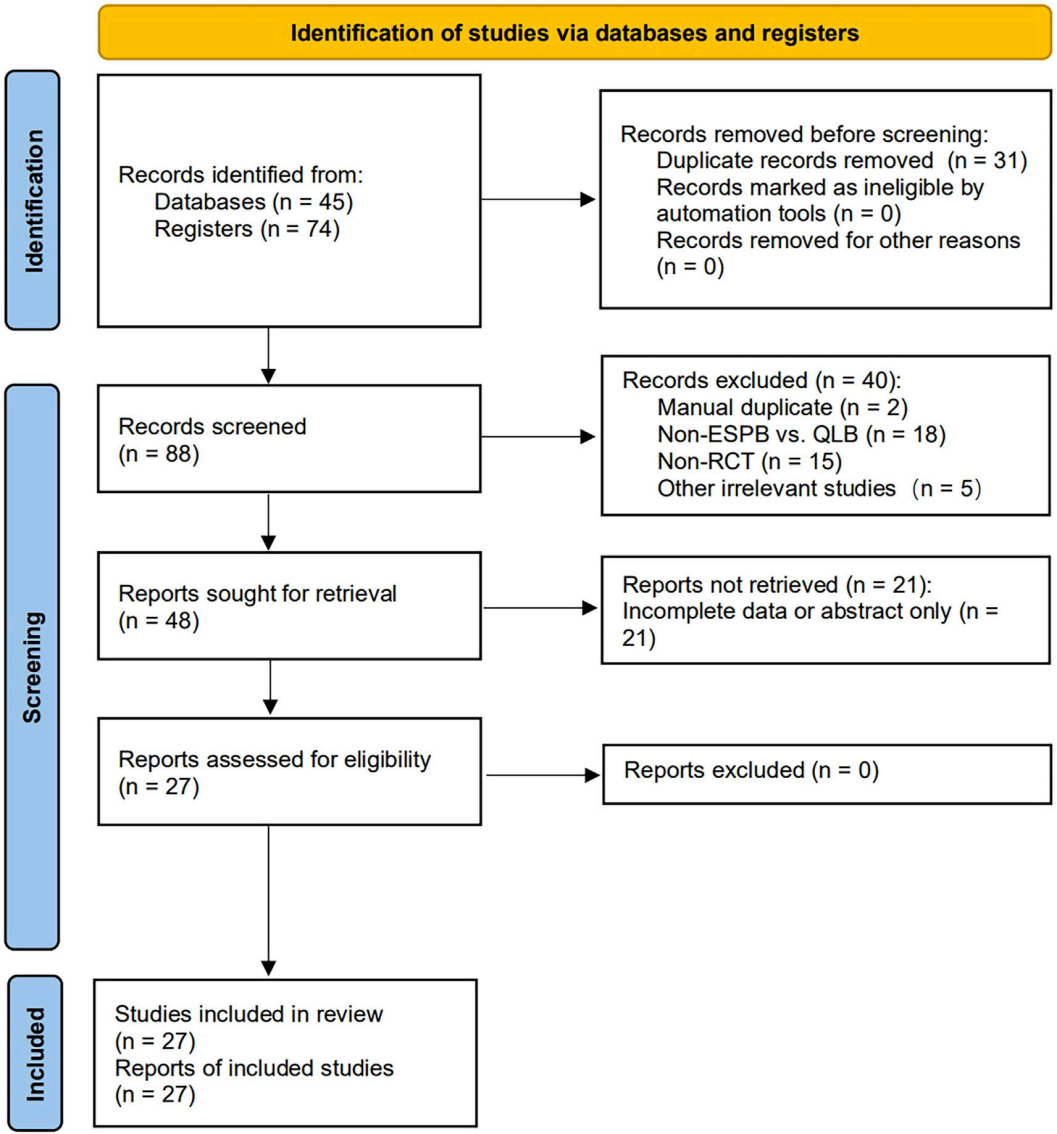
Flow diagram of study selection.

A total of 119 articles for possible inclusion were identified from the initial search of the four databases. After removing duplicates via EndNote V.20. Software (Clarivate Analytics, United States, 2021) and screening by reading titles and abstracts, we reviewed the full texts of 48 potentially relevant studies. Ultimately, this meta-analysis included 27 RCTs that were published between 2018 and 2024 (Mahmoud Fakhry et al., 2024; Hassanein et al., 2023; Barthwal et al., 2023; Taman et al., 2022; Mohasseb et al., 2021; Abd Ellatif and Abdelnaby, 2021; Abdelaziz et al., 2024; Zhang et al., 2024; Onay et al., 2023; Tulgar et al., 2018; Aksu et al., 2019; Aygun et al., 2020; Jared et al., 2020; Kang et al., 2021; Srivastawa et al., 2022; Joshi et al., 2022; Aksoy et al., 1992; Ashoor et al., 2023; Baran, 2023; Jiang et al., 2023; Kumar and Raj, 2023; Mostafa et al., 2023; Priya et al., 2023; Joshi et al., 2024; Ralte et al., 2024; Saini et al., 2024; Seif et al., 2024). The study selection process is depicted in the detailed PRISMA flow diagram in Figure 1.

### 3.2 Study characteristics and bias

We outline the baseline characteristics of the 27 RCTs included in this study in Table 2. Twenty-seven RCTs included 1942 patients who received either QLB or ESPB during different surgeries. Among these 27 studies, seventeen were from Asia (China, India, Turkey and Korea) (Barthwal et al., 2023; Zhang et al., 2024; Onay et al., 2023; Tulgar et al., 2018; Aksu et al., 2019; Aygun et al., 2020; Kang et al., 2021; Srivastawa et al., 2022; Joshi et al., 2022; Aksoy et al., 1992; Baran, 2023; Jiang et al., 2023; Kumar and Raj, 2023; Priya et al., 2023; Joshi et al., 2024; Ralte et al., 2024; Saini et al., 2024), nine from Africa (Egypt) (Mahmoud Fakhry et al., 2024; Hassanein et al., 2023; Taman et al., 2022; Mohasseb et al., 2021; Abd Ellatif and Abdelnaby, 2021; Abdelaziz et al., 2024; Ashoor et al., 2023; Mostafa et al., 2023; Seif et al., 2024), and one from North America (United States) (Jared et al., 2020). Patients were aged between 1 and 7 years or between 18 and 85 years. After bias assessment with the Cochrane Tool, most studies had a low risk of bias, except for eight (Hassanein et al., 2023; Barthwal et al., 2023; Taman et al., 2022; Abd Ellatif and Abdelnaby, 2021; Jared et al., 2020; Joshi et al., 2022; Kumar and Raj, 2023; Saini et al., 2024) with unclear randomization and six (Mahmoud Fakhry et al., 2024; Barthwal et al., 2023; Joshi et al., 2022; Kumar and Raj, 2023; Priya et al., 2023; Saini et al., 2024) in the measurement of the outcome.

**TABLE 2:** Characteristics of the included studies.

Study	Country	Sample	Age (yr)	ASA	Type of surgery	NB method	NB drug	NB volume	Outcomes
Isaac Lalfakzuala Ralte-2024	India	60	1–6	I-II	Elective open pyeloplasty	ESPB (n = 30), QLB (n = 30)	Ropivacaine	0.25%, 0.5 mL/kg	a, b, c, d, e, g, h, i, r
Can Aksu-2019	Turkey	57	1–7	I-II	Lower abdominal surgery	ESPB (n = 28), QLB (n = 29)	Bupivacaine	0.25%, 0.5 mL/kg	b, c, g, h, i
Hani I. Taman-2022	Egypt	85	2–7	NR	Laparoscopic abdominal surgery	ESPB (n = 43), QLB (n = 42)	Bupivacaine	0.25%, 0.5 mL/kg	b, c, d, e, f, g, i, o, p
Ahmed Hassanein-2023	Egypt	40	18–70	I-II	Laparoscopic cholecystectomy	ESPB (n = 20), QLB (n = 20)	Bupivacaine	0.25%, 20 mL	b, c, d, e, h, k, o, r
Hakan Aygun-2020	Turkey	80	18–70	I-II	Laparoscopic cholecystectomy	ESPB (n = 40), QLB (n = 40)	Bupivacaine + Lidocaine	0.5%, 30 mL + 2%, 10 mL	a, c, d, e, f, g, h, m
Jared A. Herman-2020	United States	80	NR	NR	Laparoscopic cholecystectomy	ESPB (n = 40), QLB (n = 40)	NR	NR	a, c, d, e, h
RyungA Kang-2021	Korea	85	40–65	I-III	Laparoscopic liver resection	ESPB (n = 42), QLB (n = 43)	Ropivacaine	0.375%, 20 mL	a, e, f, h, i, m, q, s
Tarek M Ashoor-2023	Egypt	66	21–60	NR	Laparoscopic sleeve gastrectomy	ESPB (n = 32), QLB (n = 34)	Bupivacaine	0.25%, 30 mL	a, b, c, d, e, f, g, h, i, k, n, p, r, t, u
Ahmed M. Mohasseb-2021	Egypt	68	40–60	I-II	Open colorectal cancer surgery	ESPB (n = 34), QLB (n = 34)	Bupivacaine	0.25%, 20 mL	a, b, c, e, h, k, m
Dina Mahmoud Fakhry-2024	Egypt	60	35–75	I-III	Laparoscopic resection of colorectal cancer	ESPB (n = 30), QLB (n = 30)	Bupivacaine	0.25%, 20 mL	a, b, c, d, e, f, h, i, q
Serkan Tulgar-2018	Turkey	40	18–85	I-III	Hip and proximal femur surgery	ESPB (n = 20), QLB (n = 20)	Bupivacaine + Lidocaine	0.5%, 20 mL + 2%, 10 mL	a, c, d, e, f, g, h, m, n
Nazmy Edward Seif-2024	Egypt	80	18–70	II-III	Radical cystectomy	ESPB (n = 40), QLB (n = 40)	Bupivacaine	0.25%, 30 mL	a, b, c, d, e, f, h, r
Apoorva Bakshi-2022	India	60	20–40	II	Cesarean section	ESPB (n = 30), QLB (n = 30)	Ropivacaine	0.375%, 20 mL	a, b, c, d, e, h, i, k
Joshi R-2022	India	112	18–40	II	Cesarean section	ESPB (n = 56), QLB (n = 56)	Ropivacaine	0.25%, 20 mL	b, c, d, e, h, i, m
Joshi R-2024	India	112	18–40	II	Cesarean section	ESPB (n = 56), QLB (n = 56)	Ropivacaine	0.25%, 20 mL	a, b, c, d, e, h, i, m
Maha Mostafa-2023	Egypt	101	18–35	II	Cesarean section	ESPB (n = 50), QLB (n = 51)	Bupivacaine	0.25%, 20 mL	a, b, d, e, g, h, i, q, t
Mehmet Aksoy-2023	Turkey	40	18–45	II	Cesarean section	ESPB (n = 20), QLB (n = 20)	Bupivacaine	0.25%, 20 mL	a, b, c, d, e, h, t
Ravi Kumar-2023	India	100	25–35	I-II	Cesarean section	ESPB (n = 50), QLB (n = 50)	Bupivacaine	0.25%, 20 mL	d, e, r
T. K Priya-2023	India	52	18–40	II-IIE	Cesarean section	ESPB (n = 26), QLB (n = 26)	Bupivacaine	0.25%, 0.3 mL/kg	a, c, d, e, h, q
Preeti Saini-2024	India	126	18–60	I-II	Percutaneous nephrolithotomy	ESPB (n = 63), QLB (n = 63)	Ropivacaine	0.25%, 15 mL	a, b, c, d, e, f, h
Abd Ellatif SE-2021	Egypt	50	21–60	II-III	Open nephrectomy	ESPB (n = 25), QLB (n = 25)	Bupivacaine	0.25%, 0.3–0.4 mL/kg	a, b, c, d, e, f, h, r, u
Meryem ONAY-2023	Turkey	40	18–70	I-III	Open nephrectomy	ESPB (n = 20), QLB (n = 20)	Bupivacaine	0.25%, 20 mL	a, c, d, e, h, m, p, r
Zhen Zhang-2024	China	110	18–70	I-III	Laparoscopic nephrectomy	ESPB (n = 55), QLB (n = 55)	Ropivacaine	0.375%, 0.4 mL/kg	a, c, d, e, g, h, m, q, s, u
Abhilasha Barthwal-2023	India	60	35–65	I-II	Total abdominal hysterectomy	ESPB (n = 30), QLB (n = 30)	Ropivacaine	0.375%, 20 mL	a, b, c, d, e, h, i
O Baran-2023	Turkey	54	18–75	I-II	Laparoscopic hysterectomy	ESPB (n = 27), QLB (n = 27)	Bupivacaine	0.25%, 30 mL	a, b, c, d, e, f, h, r
TSA Abdelaziz-2024	Egypt	64	40–60	I-II	Abdominal hysterectomy	ESPB (n = 32), QLB (n = 32)	Bupivacaine	0.25%, 20 mL	a, b, c, d, e, f, h, r
Weiwei Jiang-2023	China	60	18–65	I-II	Total laparoscopic hysterectomy	ESPB (n = 30), QLB (n = 30)	Ropivacaine	0.4%, 25 mL	a, b, c, d, e, h, j, m, q, t, u

a. Postoperative analgesic consumption over 24 h.

b. Time to the first analgesic request.

c. Postoperative pain scores over 6 h.

d. Postoperative pain scores over 12 h.

e. Postoperative pain scores over 24 h.

f. Block performance time.

g. Postoperative rescue analgesia rate.

h. Incidence of complications.

i. Postoperative satisfaction (rate).

j. Postoperative chronic pain score.

k. Duration of analgesia.

l. Intraoperative fentanyl dosage.

m. Postoperative analgesic consumption at other times.

n. Dose to analgesic request.

o. Mean dose of postoperative fentanyl.

p. Number of cases of postoperative pain rescue.

q. Postoperative recovery quality score.

r. Hemodynamic parameters.

s. Sleep quality on the first night.

t. Time to first ambulation.

u. Postoperative length of hospitalization.

Abbreviations: ASA, american society of anesthesiologists; NB, nerve block; ESPB, erector spinae plane block; QLB, quadratus lumborum block; NR, not reported.

### 3.3 Meta-analysis of primary outcomes

#### 3.3.1 Postoperative analgesic consumption over 24 h

**FIGURE 2:**
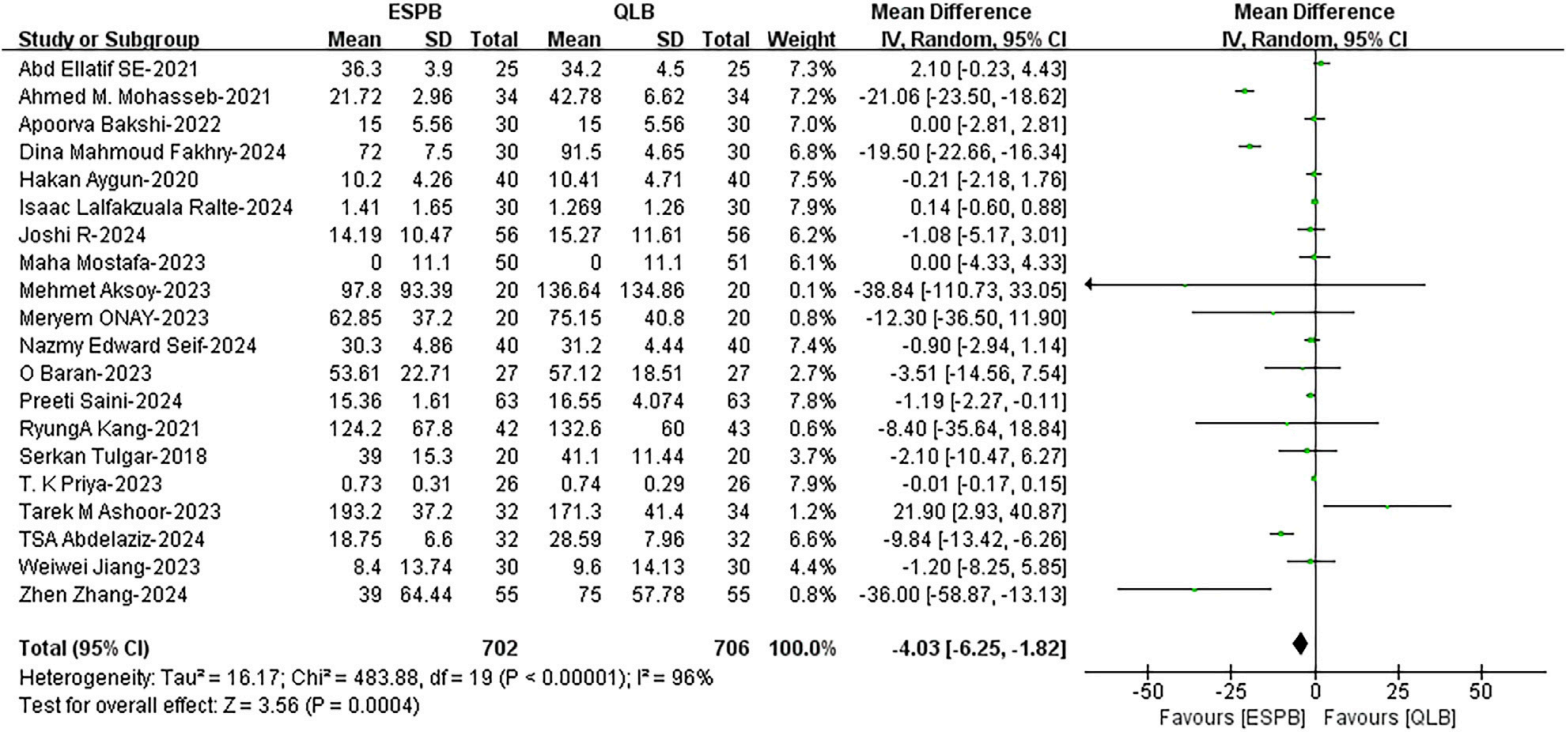
Postoperative analgesic consumption over 24 h between ESPB and QLB. ESPB, erector spinae plane block; QLB, quadratus lumborum block; SD, standard deviation; IV, inverse variance method; CI, confidence interval.

A comprehensive analysis of twenty studies (Mahmoud Fakhry et al., 2024; Mohasseb et al., 2021; Abd Ellatif and Abdelnaby, 2021; Abdelaziz et al., 2024; Zhang et al., 2024; Onay et al., 2023; Tulgar et al., 2018; Aygun et al., 2020; Kang et al., 2021; Srivastawa et al., 2022; Aksoy et al., 1992; Ashoor et al., 2023; Baran, 2023; Jiang et al., 2023; Mostafa et al., 2023; Priya et al., 2023; Joshi et al., 2024; Ralte et al., 2024; Saini et al., 2024; Seif et al., 2024) revealed that, compared with QLB, ESPB consumed fewer analgesics over 24 h postoperatively (WMD, −4.03; 95% confidence interval (CI), −6.25 to −1.82; I^2^ = 96%; *P* = 0.0004; moderate quality of evidence) (Figure 2). The subgroup analysis for blocking drug revealed that heterogeneity originated mainly from the bupivacaine group, and there might be high clinical or methodological heterogeneity within the group (Supplementary Figure S1A). Surgery type was not the main source of heterogeneity (Supplementary Figure S1B), suggesting that this result is applicable to different types of surgery. The robustness of these results was confirmed by a sensitivity analysis (Supplementary Figure S2).

### 3.4 Meta-analysis of secondary outcomes

#### 3.4.1 Time to the first analgesic request

**FIGURE 3:**
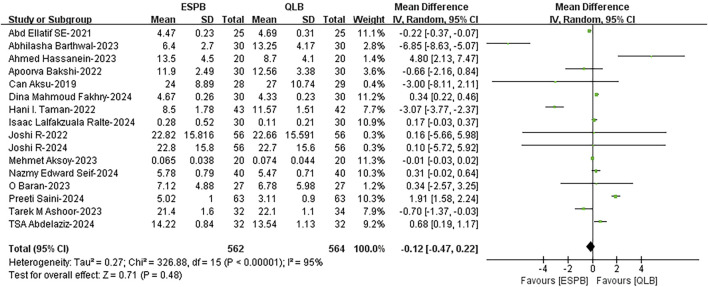
Time to the first analgesic request between ESPB and QLB. ESPB, erector spinae plane block; QLB, quadratus lumborum block; SD, standard deviation; IV, inverse variance method; CI, confidence interval.

The time to the first analgesic request was reported in sixteen included studies (Mahmoud Fakhry et al., 2024; Hassanein et al., 2023; Barthwal et al., 2023; Taman et al., 2022; Abd Ellatif and Abdelnaby, 2021; Abdelaziz et al., 2024; Aksu et al., 2019; Srivastawa et al., 2022; Joshi et al., 2022; Aksoy et al., 1992; Ashoor et al., 2023; Baran, 2023; Joshi et al., 2024; Ralte et al., 2024; Saini et al., 2024; Seif et al., 2024). Data analysis revealed that there was no significant difference between the ESPB and QLB groups (weighted mean difference (WMD), −0.12; 95% CI, −0.47 to 0.22; I^2^ = 95%; *P* = 0.48; moderate quality of evidence) (Figure 3). In the subgroup analysis for age and blocking drug, the results indicated that none of these factors were the main source of heterogeneity (Supplementary Figures S3A, S3B), indicating that these results are applicable to different ages and blocking drugs. The subgroup analysis results of the surgery type revealed that the heterogeneity originated mainly from the kidney surgery group, and there might be high clinical or methodological heterogeneity within the group (Supplementary Figure S3C). The source of heterogeneity was not found after sensitivity analysis by eliminating individual studies and observing whether the heterogeneity was reduced (Supplementary Figure S4).

#### 3.4.2 Postoperative resting pain scores

**FIGURE 4:**
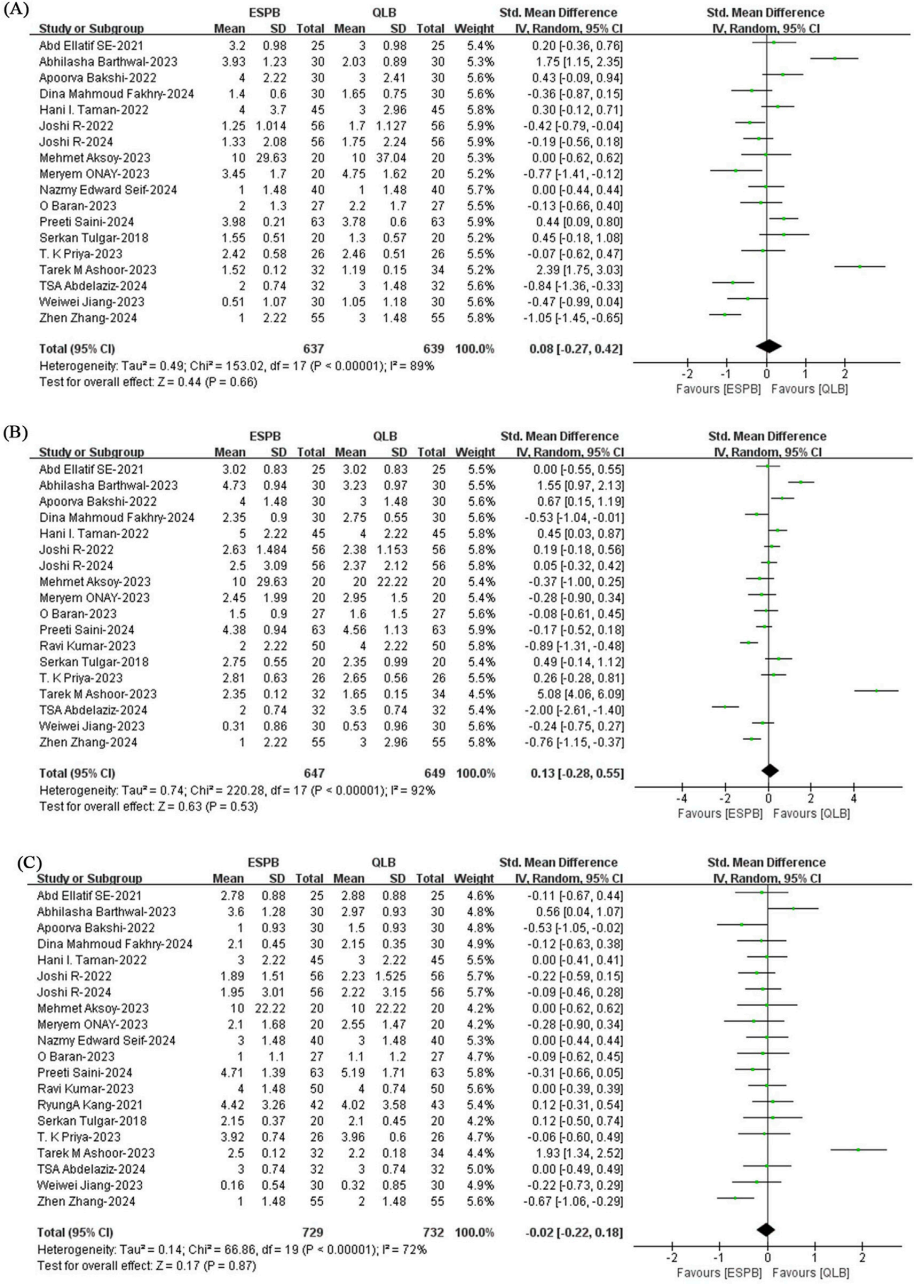
Postoperative resting pain scores at **(A)** 6 h, **(B)** 12 h and **(C)** 24 h for the ESPB and QLB groups. ESPB, erector spinae plane block; QLB, quadratus lumborum block; SD, standard deviation; IV, inverse variance method; CI, confidence interval.

The combined results of twenty studies (Mahmoud Fakhry et al., 2024; Barthwal et al., 2023; Taman et al., 2022; Abd Ellatif and Abdelnaby, 2021; Abdelaziz et al., 2024; Zhang et al., 2024; Onay et al., 2023; Tulgar et al., 2018; Kang et al., 2021; Srivastawa et al., 2022; Joshi et al., 2022; Aksoy et al., 1992; Ashoor et al., 2023; Baran, 2023; Jiang et al., 2023; Kumar and Raj, 2023; Priya et al., 2023; Joshi et al., 2024; Saini et al., 2024; Seif et al., 2024) showed that the postoperative resting pain scores at 6 h, 12 h and 24 h in the QLB group were similar to those in the ESPB group (6 h: SMD, 0.08; 95% CI, −0.27 to 0.24; I^2^ = 89%; *P* = 0.66; moderate quality of evidence; 12 h: SMD, 0.13; 95% CI, −0.28 to 0.55; I^2^ = 92%; *P* = 0.53; moderate quality of evidence; 24 h: SMD, −0.02; 95% CI, −0.22 to 0.18; I^2^ = 72%; *P* = 0.87; moderate quality of evidence) (Figures 4A–C). The subgroup analysis did not significantly reduce heterogeneity, so neither the blocking drug nor the type of surgery was a major source of heterogeneity in the postoperative resting pain scores at 6 h and 12 h (Supplementary Figures S5A, S5B; S6A, S6B). The subgroup analysis results for blocking drug for postoperative 24-h resting pain scores showed that blocking drug was not the main source of heterogeneity (Supplementary Figure S7A), but the surgery type affected the results (Supplementary Figure S7B). The stability of the results for the 6-h and 12-h postoperative resting pain scores was confirmed by sensitivity analysis (Supplementary Figures S8, S9). The I^2^ value decreased from 72% to 18% after the removal of one study reported by Ashoor et al. (2023), suggesting that the RCT caused significant heterogeneity in the analysis of the 24-h postoperative resting pain score (Supplementary Figure S10).

#### 3.4.3 Block performance time

**FIGURE 5:**
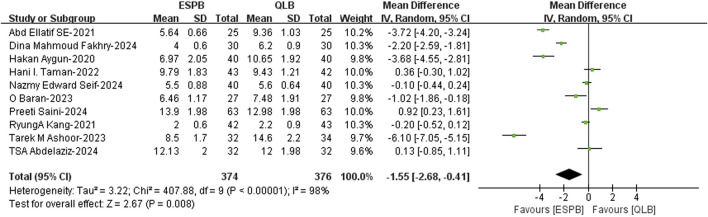
Block performance time comparison of ESPB and QLB. ESPB, erector spinae plane block; QLB, quadratus lumborum block; SD, standard deviation; IV, inverse variance method; CI, confidence interval.

The pooled data analysis from ten studies (Mahmoud Fakhry et al., 2024; Taman et al., 2022; Abd Ellatif and Abdelnaby, 2021; Abdelaziz et al., 2024; Aygun et al., 2020; Kang et al., 2021; Ashoor et al., 2023; Baran, 2023; Saini et al., 2024; Seif et al., 2024) revealed that the block performance time of ESPB was shorter than that of QLB (WMD, −1.55; 95% CI, −2.68 to −0.41; I^2^ = 98%; *P* = 0.008; moderate quality of evidence) (Figure 5). The robustness of these studies was confirmed by a sensitivity analysis (Supplementary Figure S11).

#### 3.4.4 Postoperative rescue analgesia rate

**FIGURE 6:**
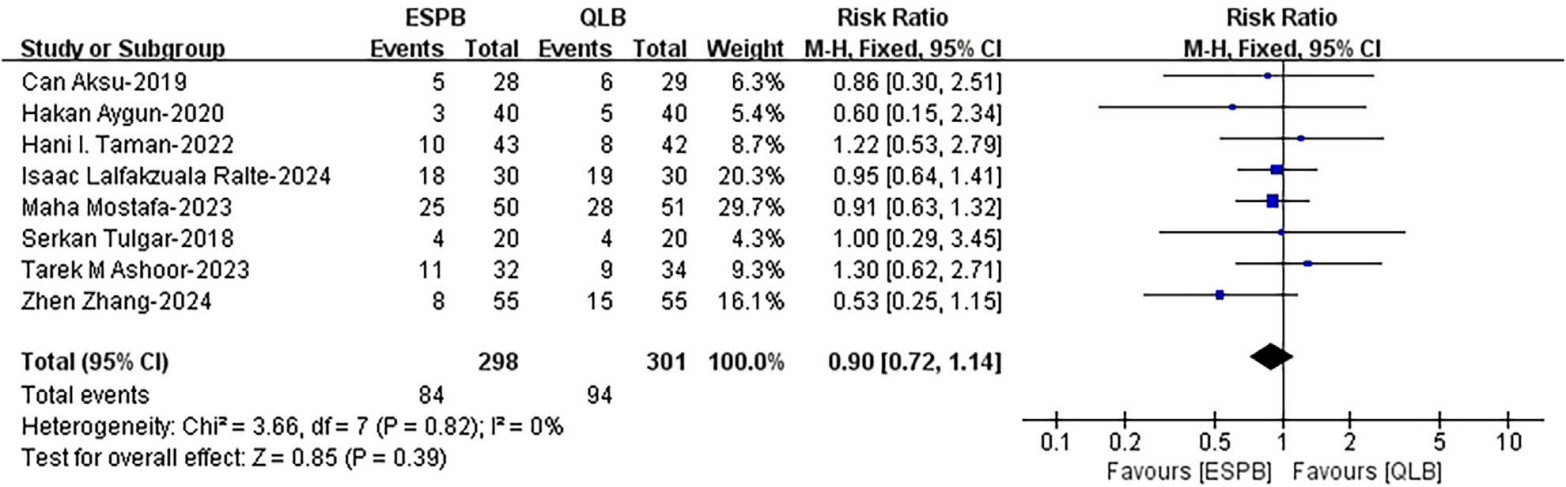
Comparison of the postoperative rescue analgesia rates between ESPB and QLB. ESPB, erector spinae plane block; QLB, quadratus lumborum block; H-M, Mantel - Haenszel method; CI, confidence interval.

The outcome was reported in eight included studies (Taman et al., 2022; Zhang et al., 2024; Tulgar et al., 2018; Aksu et al., 2019; Aygun et al., 2020; Ashoor et al., 2023; Mostafa et al., 2023; Ralte et al., 2024). Data analysis revealed no significant difference in the postoperative rescue analgesia rate between the ESPB group and the QLB group (risk ratio (RR), 0.90; 95% CI, 0.72 to 1.14; I^2^ = 0%; *P* = 0.39; high quality of evidence) (Figure 6). In the subgroup analysis for age, the results demonstrated that age had no effect on the results (Supplementary Figure S12). The stability of these results was confirmed by sensitivity analysis (Supplementary Figure S13).

#### 3.4.5 Incidence of complications

**FIGURE 7:**
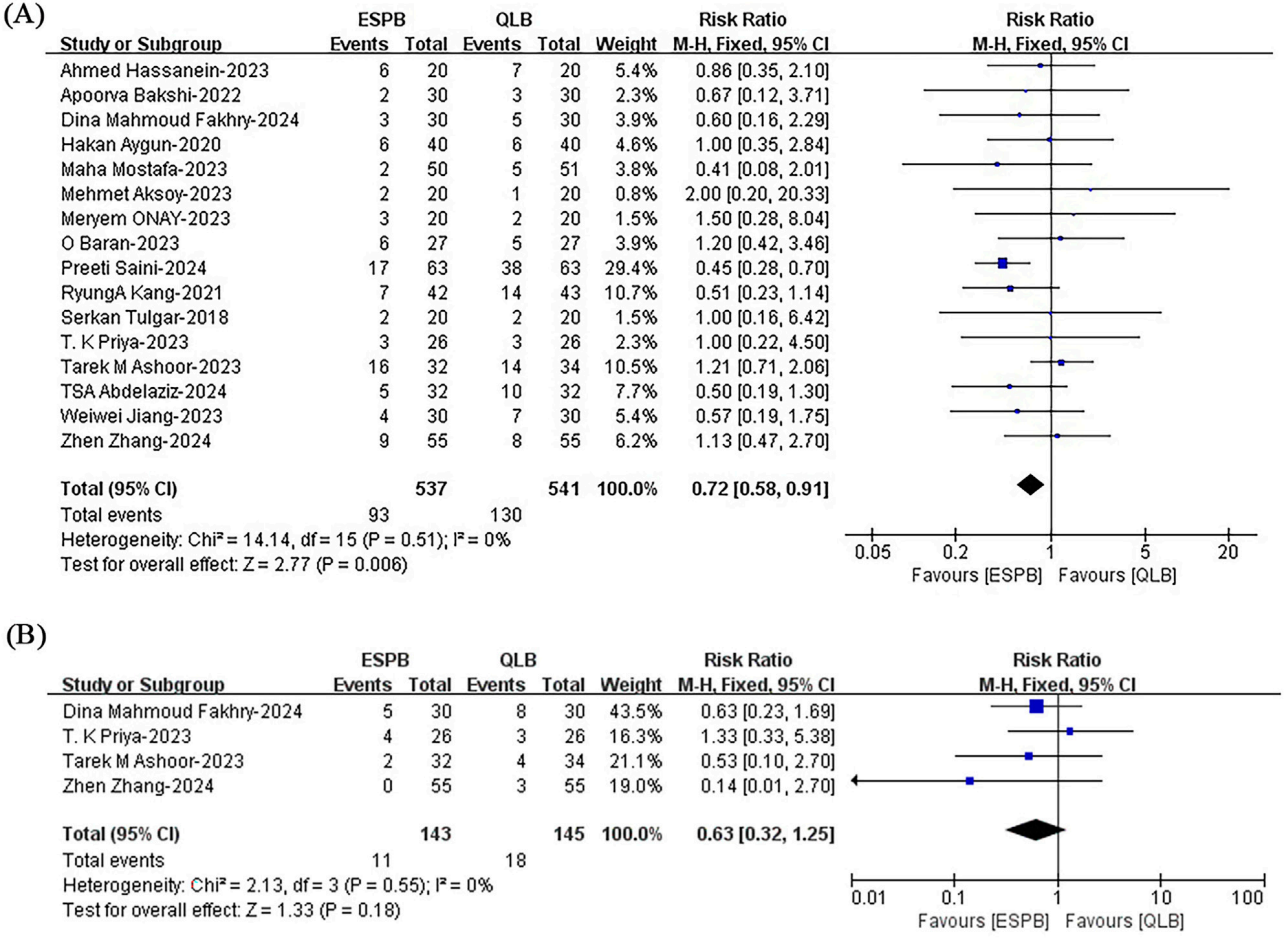
Incidence of postoperative **(A)** nausea and vomiting and **(B)** hypotension between ESPB and QLB. ESPB, erector spinae plane block; QLB, quadratus lumborum block; H-M, Mantel - Haenszel method; CI, confidence interval.

Pooled results from sixteen studies (Mahmoud Fakhry et al., 2024; Hassanein et al., 2023; Abdelaziz et al., 2024; Onay et al., 2023; Tulgar et al., 2018; Aygun et al., 2020; Kang et al., 2021; Srivastawa et al., 2022; Aksoy et al., 1992; Ashoor et al., 2023; Baran, 2023; Jiang et al., 2023; Mostafa et al., 2023; Priya et al., 2023; Saini et al., 2024; Yang et al., 2023) revealed a lower incidence of postoperative nausea and vomiting with ESPB than with QLB (RR, 0.72; 95% CI, 0.58 to 0.91; I^2^ = 0%; *P* = 0.006; high quality of evidence) (Figure 7A). The subgroup analysis results for blocking drug and surgery type indicated that these two factors affected the results (Supplementary Figures S14A, S14B). More evidence is needed to support this conclusion. There was no significant difference between ESPB and QLB for postoperative hypotension (RR, 0.63; 95% CI, 0.32 to 1.25; I^2^ = 0%; *P* = 0.18; moderate quality of evidence) (Figure 7B). The robustness of these studies was confirmed by a sensitivity analysis (Supplementary Figures S15, S16).

#### 3.4.6 Postoperative satisfaction

**FIGURE 8:**
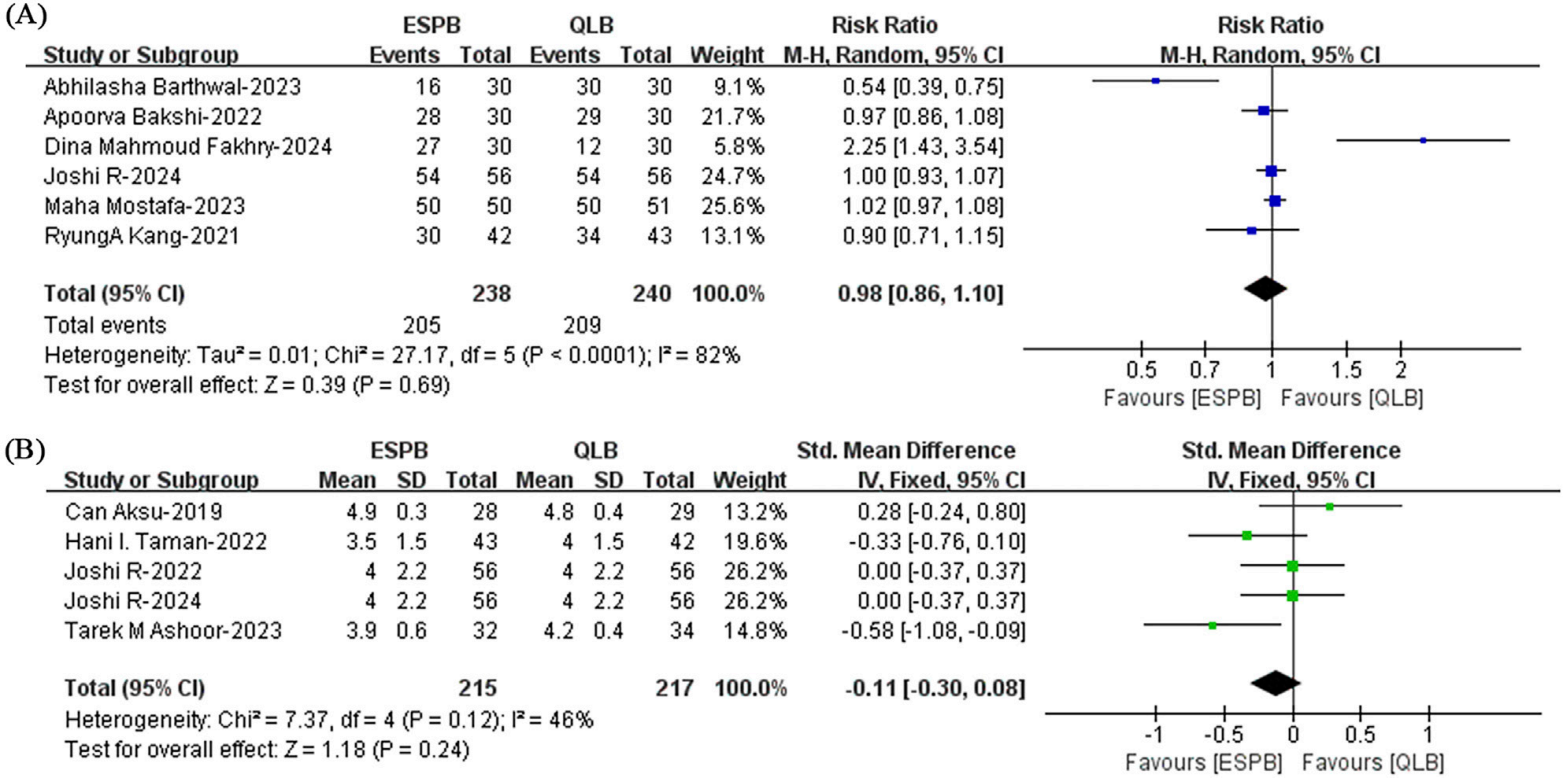
**(A)** Comparison of the postoperative satisfaction rate and **(B)** postoperative satisfaction between the ESPB and QLB groups. ESPB, erector spinae plane block; QLB, quadratus lumborum block; H - M, Mantel - Haenszel method; CI, confidence interval; SD, standard deviation; IV, inverse variance method.

A total of ten studies (Mahmoud Fakhry et al., 2024; Barthwal et al., 2023; Taman et al., 2022; Aksu et al., 2019; Kang et al., 2021; Srivastawa et al., 2022; Joshi et al., 2022; Ashoor et al., 2023; Mostafa et al., 2023; Joshi et al., 2024) discussed this issue. A comprehensive analysis revealed no significant difference in the postoperative satisfaction rate (RR, 0.98; 95% CI, 0.86 to 1.10; I^2^ = 82%; *P* = 0.69; moderate quality of evidence) or postoperative satisfaction (SMD, −0.11; 95% CI, −0.30 to 0.08; I^2^ = 46%; *P* = 0.24; moderate quality of evidence) between the ESPB and QLB groups (Figure 8). The robustness of the results for the satisfaction rate was confirmed by sensitivity analysis (Supplementary Figure S17). The I^2^ value decreased from 46% to 9% after the removal of one study reported by Ashoor et al. (2023), which showed that the study caused significant heterogeneity in the analysis of postoperative satisfaction (Supplementary Figure S18).

### 3.5 Publication bias

Supplementary Figures S19A–S19G shows the funnel plots for postoperative analgesic consumption over 24 h, time to the first analgesic request, postoperative pain scores at 6, 12 and 24 h, block performance time and incidence of postoperative nausea and vomiting, and all the funnel plots displayed some symmetry. Supplementary Materia1 S3 shows the results of the Begg test for these outcomes, and all *P* values were >0.05. Thus, the above analysis suggested a low level of risk of publication bias for these outcomes.

## 4 Discussion

Taken together, the following points can be concluded. First, in terms of postoperative analgesic consumption over 24 h, moderate-quality evidence indicated that ESPB consumed fewer analgesics postoperatively and provided better analgesia than QLB. Second, moderate-quality evidence showed that the ESPB and QLB had similar effects on the time to the first analgesic request and postoperative resting pain scores. Third, moderate-quality evidence revealed that the performance time of ESPB was shorter than that of the QLB. High-quality evidence showed that ESPB has a lower incidence of postoperative nausea and vomiting. Finally, moderate-to high-evidence showed that ESPB and QLB showed comparable effects on postoperative rescue analgesia and patient satisfaction.

Currently, ultrasound-guided ESPB and QLB are commonly used in abdomen and proximal lower extremity surgeries, such as laparoscopic cholecystectomy, laparoscopic resection of colorectal cancer, laparoscopic hysterectomy and cesarean section for intraoperative and postoperative analgesia. Two previous meta-analyses compared the analgesic effects of ESPB with those of QLB in laparoscopic surgeries. One study (Yang et al., 2023) reported no significant difference in postoperative analgesic consumption over 24 h or the incidence of complications between ESPB and QLB in adults undergoing laparoscopic cholecystectomy. Another study (Oraee et al., 2024) showed that, compared with QLB, ESPB had similar 24-h postoperative analgesic consumption, higher 24-h postoperative resting pain scores, a shorter time to the first analgesic request, and fewer postoperative complications in laparoscopic surgeries. In the present meta-analysis, we did not restrict the type of surgery and included more studies. The results revealed lower 24-h analgesic consumption after surgery in the ESPB group than in the QLB group. In the forest plot, four studies (Mahmoud Fakhry et al., 2024; Mohasseb et al., 2021; Abdelaziz et al., 2024; Zhang et al., 2024) supported ESPB, and one study (Ashoor et al., 2023) supported the QLB. Most studies reported less consumption with ESPB, but the difference was not statistically significant. The sensitivity analysis revealed that the results were robust. Interestingly, our results showed that there was no difference in the postoperative resting pain score between ESPB and QLB at 6 h, 12 h, or 24 h, indicating that ESPB has the same analgesic effect as QLB. However, this conclusion might due to that more analgesics were used in the QLB group, resulting in no significant difference in postoperative pain scores between the two groups. The pooled results of the ten included studies showed that the performance time of ESPB was shorter than that of the QLB. In the forest plot, five studies (Mahmoud Fakhry et al., 2024; Abd Ellatif and Abdelnaby, 2021; Aygun et al., 2020; Ashoor et al., 2023; Baran, 2023) supported ESPB, one study (Saini et al., 2024) supported the QLB, and four studies showed no statistical significance. Moreover, the sensitivity analysis results showed that the results were robust. Therefore, this meta-analysis revealed that, compared with QLB, ESPB is easier to perform and results in less analgesic consumption.

In general, the sources of heterogeneity of the included studies included statistical heterogeneity, clinical heterogeneity, and methodological heterogeneity. In the present study, some outcomes remained highly heterogeneous after subgroup analysis (age, blocking drug and type of surgery), and a random-effect model and sensitivity analysis were performed to reduce heterogeneity. One of the possible reasons is the high degree of clinical heterogeneity among these studies due to the large differences in patient and anesthesia type, local anesthetic dose and concentration, surgical incision size and location, and patient tolerance for pain. In subgroup analysis, the correlation between subgroup factors and conclusions can be analyzed when heterogeneity is not significant. In this study, the incidence of postoperative nausea and vomiting before subgroup analysis had low heterogeneity (I^2^ = 0%) and *P* = 0.006, indicating high consistency among the included studies. After subgroup analysis based on blocking drugs, the *P* value of the ropivacaine subgroup was 0.04, which may indicate that the blocking drug had a significant impact on the results. However, subgroup analyses are usually exploratory rather than confirmatory, and even the *P* value of the ropivacaine group of 0.04 did not indicate an impact on the results. More evidence is needed to support this conclusion.

In addition to the outcomes already stated in the results section, other relevant outcomes were reported. Two studies (Onay et al., 2023; Ashoor et al., 2023) reported no difference in postoperative analgesic demand between the ESPB and QLB groups (34.4% versus 26.5%, *P* > 0.05; 37.85 ± 29.43 versus 41.15 ± 31.75, *P* > 0.05). Five included studies (Mahmoud Fakhry et al., 2024; Zhang et al., 2024; Kang et al., 2021; Jiang et al., 2023; Priya et al., 2023) reported postoperative 24-h quality of recovery (QoR) scores. The results from three studies (Kang et al., 2021; Jiang et al., 2023; Priya et al., 2023) showed no significant difference between the ESPB and QLB groups (103.3 ± 27.1 versus 100.5 ± 32.2, *P* > 0.05; 71.0 (55.0–80.4) versus 68.3 (43.6–89.7), *P* > 0.05; 147 (Qin et al., 2024) versus 146 (Chen et al., 2021), *P* > 0.05). However, data from two other studies (Mahmoud Fakhry et al., 2024; Zhang et al., 2024) showed that the quality of recovery at 24 h after surgery was better in the ESPB group than in the QLB group (124.4 ± 12.5 versus 109.7 ± 14.6, *P* < 0.001; 113 (102–122) versus 101 (92–118), *P* < 0.05). Two included studies (Zhang et al., 2024; Kang et al., 2021) reported the quality of sleep on the first night after surgery. One study (Kang et al., 2021) used a Likert scale and reported that, compared with the QLB group, the ESPB group had a similar quality of sleep (4 (2–4.3) versus 4 (Chen et al., 2021; Kirksey et al., 2015), *P* > 0.05). However, in another study (Zhang et al., 2024), the Richards-Campbell- Sleep Questionnaire was used to measure sleep quality, and the results revealed that the ESPB group performed better (50 (33–69) versus 34 (16–54), *P* < 0.05). Four studies (Abd Ellatif and Abdelnaby, 2021; Zhang et al., 2024; Ashoor et al., 2023; Jiang et al., 2023) reported that the hospital stays (days) in the ESPB and QLB groups were similar.

To prolong the anesthetic effect and reduce the dose of local anesthetics, adjuvants such as epinephrine and dexamethasone can be added to local anesthetics to perform nerve blocks. Two included studies (Mohasseb et al., 2021; Srivastawa et al., 2022) added 4 mg dexamethasone as a local anesthetic adjuvant to ropivacaine (Srivastawa et al., 2022) or bupivacaine (Mohasseb et al., 2021) to perform ESPB and QLB. Thus, the use of dexamethasone may have influenced the results of these two studies. The results of the sensitivity analysis showed that the elimination of these two studies had no significant effect on the results. Therefore, the presence of adjuvants in local anesthetics did not affect the pooled results of the study.

This study has several limitations. First, this meta-analysis included studies involving both pediatric and adult populations, different volumes, concentrations, and types of local anesthetic agents, and various types of surgeries, which caused relatively high heterogeneity. However, subgroup analysis and sensitivity were performed to explore the source of heterogeneity and to obtain combined results for specific conditions. Second, as the approach of the QLB, surgical incision size and location and other clinical information were not described in detail in included study, the heterogeneity could not be further explored by subgroup analysis. Finally, although our results showed that 4.03 mg of OME were reduced in ESPB group compared to QLB group, whether the reduction reaches clinical significance needs to be confirmed.

## 5 Conclusion

In summary, moderate-to high-quality evidence suggested that ESPB is better than QLB for postoperative analgesia in terms of less 24-h postoperative analgesic consumption, faster block performance and a lower incidence of postoperative nausea and vomiting.

## Data Availability

The original contributions presented in the study are included in the article/Supplementary Material, further inquiries can be directed to the corresponding authors.
