# Supplementary material for: Comparison of the analgesic effects of ultrasound-guided erector spinae plane block and quadratus lumborum block: a systematic review and meta-analysis
Source: Front Pharmacol. 2025 Aug 1;16:1640135. doi: 10.3389/fphar.2025.1640135 (PMC12355214; doi:10.3389/fphar.2025.1640135)
Supplement: Supplementary file 1 [file Supplementaryfile1.docx]

**Supplementary Material 1.** Search strategies

一、Pubmed RCT-**33 articles**

(("quadratus lumborum block"[Title/Abstract]) AND (("erector spinae plane block"[Title/Abstract]) OR ("erector spinae block"[Title/Abstract])) AND ((randomized-controlled-trial[Title/Abstract]) OR (randomization[Title/Abstract]) OR (random*[Title/Abstract]) OR (controlled clinical trial[Title/Abstract]))) NOT (animals[Title/Abstract])


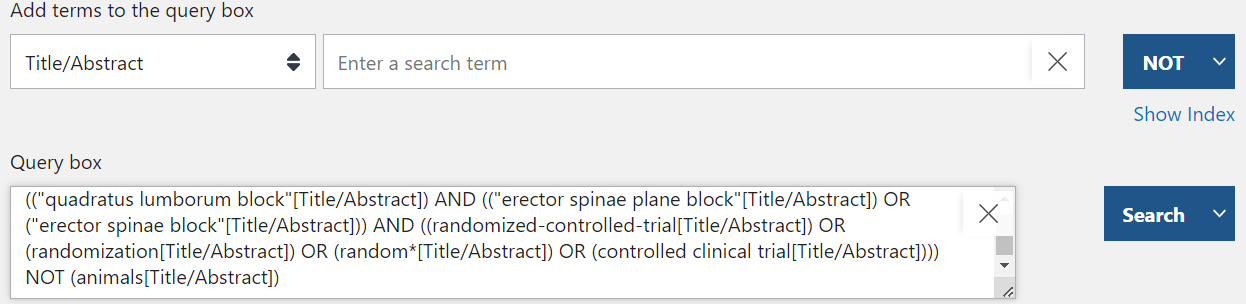


二、Cochrane library Trials-**75 articles（74 RCTs + 1 review）**


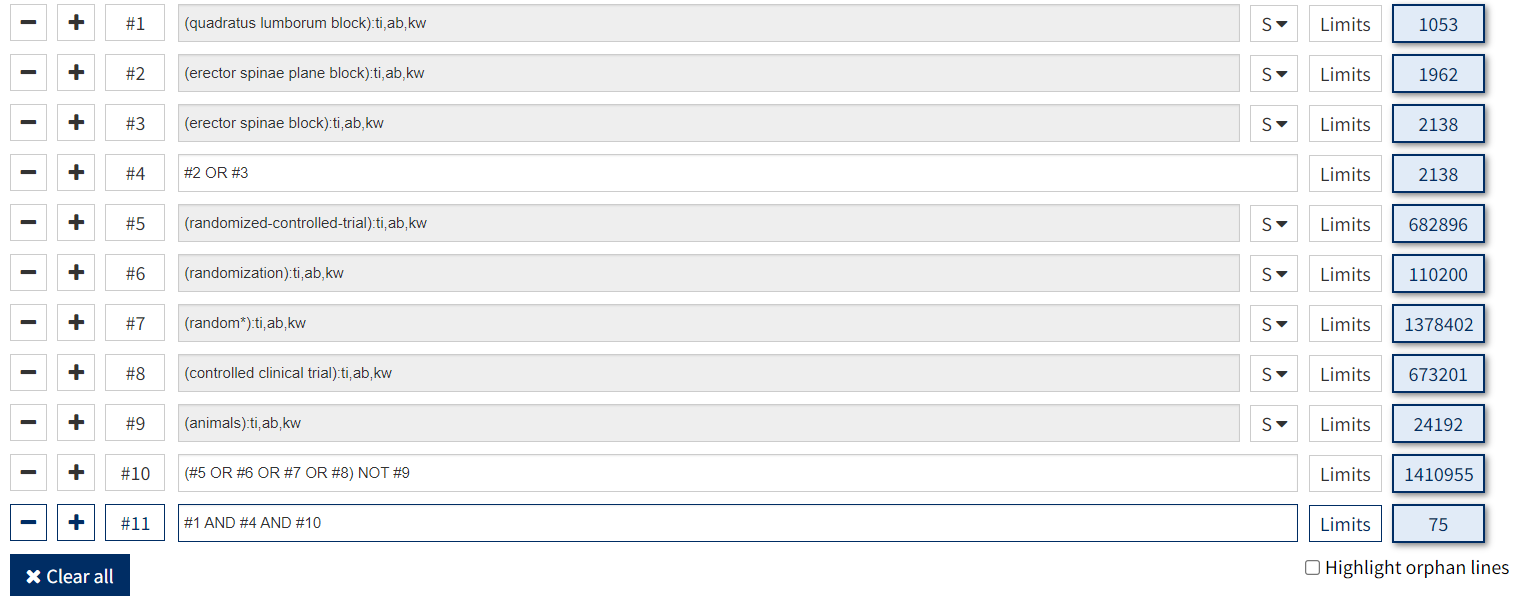


三、Embase RCT- **10 articles**


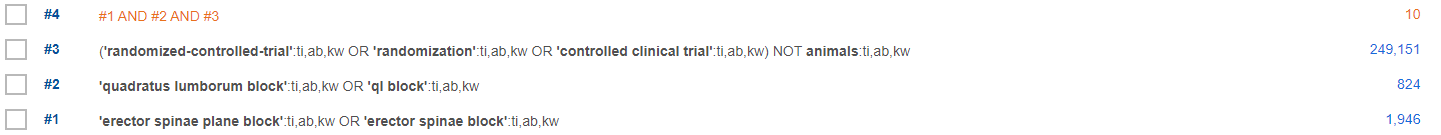


四、Web of science RCT-**2 articles**


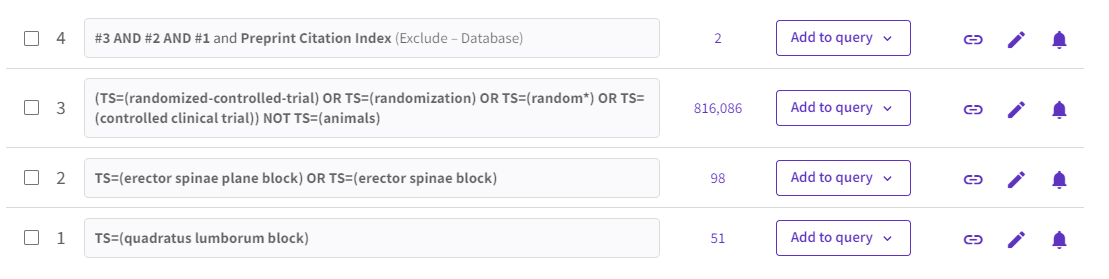


Time 2024.10.16 16:37-2024.10.16 17:59
