# Supplementary material for: Comparison of the analgesic effects of ultrasound-guided erector spinae plane block and quadratus lumborum block: a systematic review and meta-analysis
Source: Front Pharmacol. 2025 Aug 1;16:1640135. doi: 10.3389/fphar.2025.1640135 (PMC12355214; doi:10.3389/fphar.2025.1640135)
Supplement: Supplementary file 2 [file Supplementaryfile3.docx]

Supplementary Material 3. Begg test for publication bias

| Study outcome | Kendall’ score | Standard error | z value | *P* value |
| --- | --- | --- | --- | --- |
| Postoperative analgesic consumption over 24 hours | -52 | 30.822 | -1.687 | 0.092 |
| Time to the first analgesic request | -4 | 22.211 | -0.18 | 0.857 |
| Postoperative 6-h resting pain scores | 27 | 26.401 | 1.023 | 0.306 |
| Postoperative 12-h resting pain scores | 25 | 26.401 | 0.947 | 0.344 |
| Postoperative 24-h resting pain scores | 28 | 30.822 | 0.908 | 0.364 |
| Block performance time | -13 | 11.18 | -1.163 | 0.245 |
| Incidence of postoperative nausea and vomiting | 20 | 22.211 | 0.9 | 0.368 |
