# Supplementary material for: Comparison of the analgesic effects of ultrasound-guided erector spinae plane block and quadratus lumborum block: a systematic review and meta-analysis
Source: Front Pharmacol. 2025 Aug 1;16:1640135. doi: 10.3389/fphar.2025.1640135 (PMC12355214; doi:10.3389/fphar.2025.1640135)
Supplement: Supplementary file 5 [file Image1.pdf]

Supplementary Figure 1

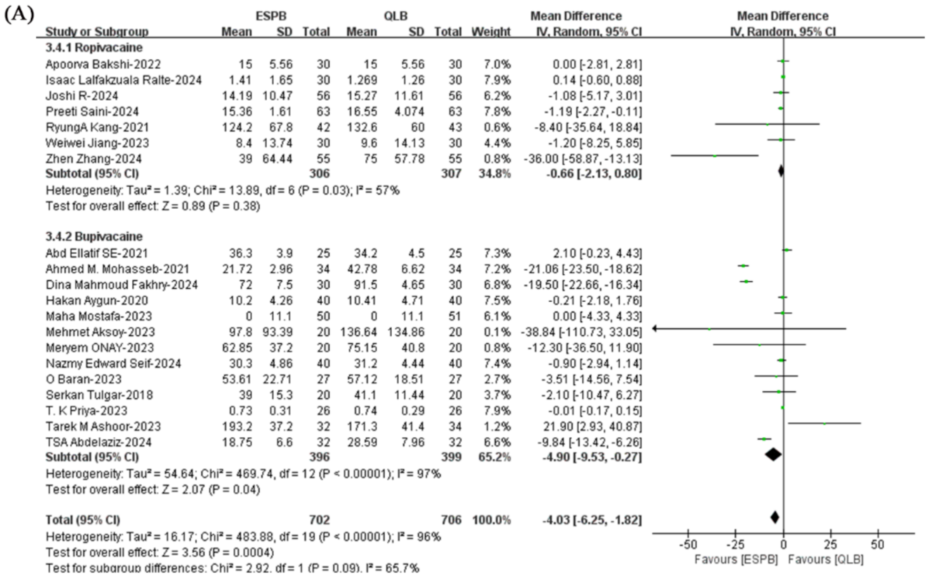

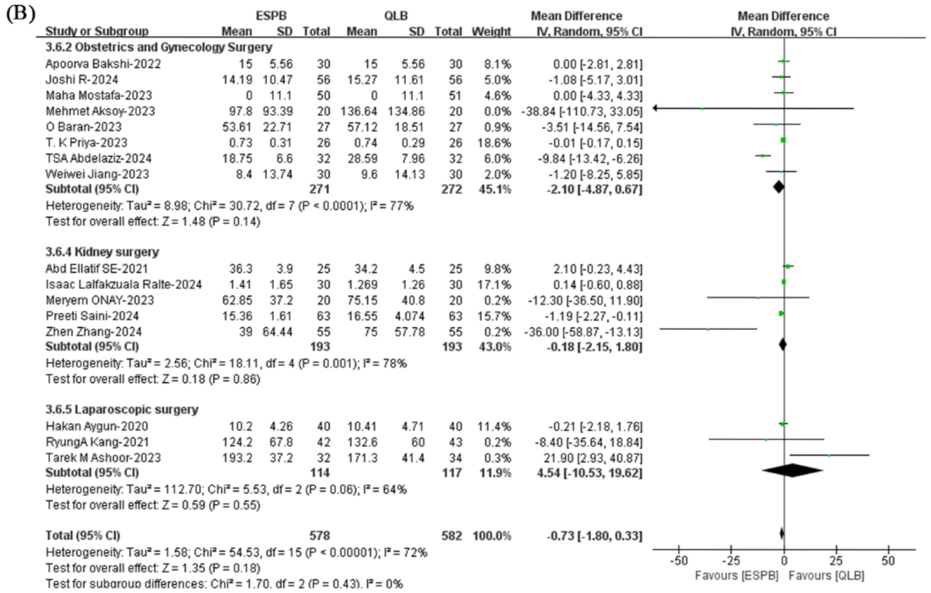

Figure S1: (A) Subgroup analysis for blocking drug of postoperative analgesic consumption over 24 hours comparing ESPB and QLB. **(B)** Subgroup analysis for surgery type of postoperative analgesic consumption over 24 hours comparing ESPB and QLB. ESPB, erector spinae plane block; QLB, quadratus lumborum block; SD, standard deviation; IV, inverse variance method; CI, confidence interval.

Supplementary Figure 2

Study	Effect Size	95% CI		P value	I^2(%)
(-)Abd Ellatif SE-2021	-0.49	-0.83	-0.15	Z = -2.820, P = 0.005	89
(-)Ahmed M. Mohasseb-2021	-0.27	-0.53	-0.02	Z = -2.085, P = 0.037	81
(-)Apoorva Bakshi-2022	-0.47	-0.82	-0.12	Z = -2.628, P = 0.009	90
(-)Dina Mahmoud Fakhry-2024	-0.31	-0.60	-0.02	Z = -2.108, P = 0.035	85
(-)Hakan Aygun-2020	-0.47	-0.82	-0.12	Z = -2.600, P = 0.009	90
(-)Isaac Lalfakzuala Ralte-2024	-0.47	-0.82	-0.13	Z = -2.663, P = 0.008	89
(-)Joshi R-2024	-0.47	-0.82	-0.11	Z = -2.561, P = 0.010	90
(-)Maha Mostafa-2023	-0.47	-0.82	-0.12	Z = -2.606, P = 0.009	89
(-)Mehmet Aksoy-2023	-0.45	-0.80	-0.10	Z = -2.540, P = 0.011	90
(-)Meryem ONAY-2023	-0.45	-0.80	-0.10	Z = -2.545, P = 0.011	90
(-)Nazmy Edward Seif-2024	-0.46	-0.81	-0.11	Z = -2.550, P = 0.011	90
(-)O Baran-2023	-0.46	-0.81	-0.11	Z = -2.576, P = 0.010	90
(-)Preeti Saini-2024	-0.45	-0.81	-0.09	Z = -2.460, P = 0.014	90
(-)RyungA Kang-2021	-0.46	-0.82	-0.11	Z = -2.567, P = 0.010	90
(-)Serkan Tulgar-2018	-0.46	-0.81	-0.11	Z = -2.590, P = 0.010	90
(-)T. K Priya-2023	-0.47	-0.81	-0.12	Z = -2.621, P = 0.009	90
(-)Tarek M Ashoor-2023	-0.50	-0.83	-0.16	Z = -2.861, P = 0.004	89
(-)TSA Abdelaziz-2024	-0.39	-0.73	-0.06	Z = -2.312, P = 0.021	89
(-)Weiwei Jiang-2023	-0.46	-0.81	-0.11	Z = -2.599, P = 0.009	90
(-)Zhen Zhang-2024	-0.44	-0.79	-0.08	Z = -2.424, P = 0.015	89
Total	-0.44	-0.78	-0.11	Z = -2.606, P = 0.009	89

Figure S2: Sensitivity analysis of postoperative analgesic consumption over 24 hours comparing ESPB and QLB. CI, confidence interval.

Supplementary Figure 3

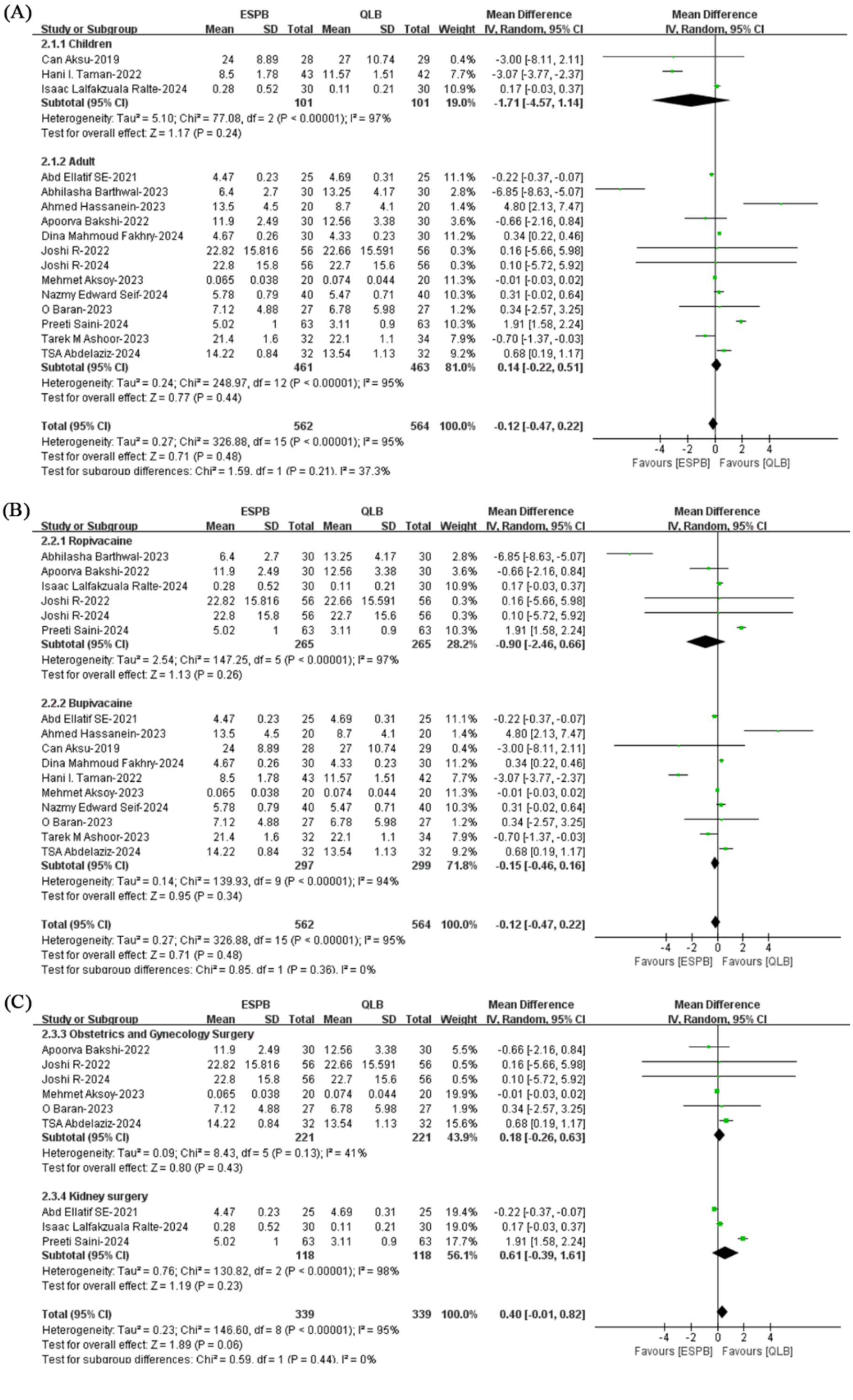

Figure S3: **(A)** Subgroup analysis for age of time to the first analgesic request comparing ESPB and QLB. **(B)** Subgroup analysis for blocking drug of time to the first

analgesic request comparing ESPB and QLB. **(C)** Subgroup analysis for surgery type of time to the first analgesic request comparing ESPB and QLB. ESPB, erector spinae plane block; QLB, quadratus lumborum block; SD, standard deviation; IV, inverse variance method; CI, confidence interval.

Supplementary Figure 4

Study	Effect Size	95% CI		P value	I^2(%)
(-)Abd Ellatif SE-2021	-0.16	-0.56	0.25	Z = -0.753, P = 0.451	96
(-)Abhilasha Barthwal-2023	0.08	-0.24	0.40	Z = 0.474, P = 0.635	95
(-)Ahmed Hassanein-2023	-0.19	-0.53	0.15	Z = -1.085, P = 0.278	96
(-)Apoorva Bakshi-2022	-0.10	-0.45	0.24	Z = -0.585, P = 0.558	96
(-)Can Aksu-2019	-0.11	-0.45	0.23	Z = -0.634, P = 0.526	96
(-)Dina Mahmoud Fakhry-2024	-0.24	-0.67	0.19	Z = -1.104, P = 0.270	95
(-)Hani I. Taman-2022	0.15	-0.17	0.47	Z = 0.923, P = 0.356	94
(-)Isaac Lalfakzuala Ralte-2024	-0.18	-0.57	0.20	Z = -0.933, P = 0.351	96
(-)Joshi R-2022	-0.13	-0.47	0.22	Z = -0.713, P = 0.476	96
(-)Joshi R-2024	-0.13	-0.47	0.22	Z = -0.712, P = 0.476	96
(-)Mehmet Aksoy-2023	-0.26	-0.78	0.26	Z = -0.989, P = 0.322	95
(-)Nazmy Edward Seif-2024	-0.18	-0.55	0.19	Z = -0.962, P = 0.336	96
(-)O Baran-2023	-0.13	-0.47	0.22	Z = -0.738, P = 0.461	96
(-)Preeti Saini-2024	-0.29	-0.59	0.01	Z = -1.886, P = 0.059	93
(-)Tarek M Ashoor-2023	-0.07	-0.43	0.28	Z = -0.407, P = 0.684	96
(-)TSA Abdelaziz-2024	-0.21	-0.57	0.15	Z = -1.122, P = 0.262	96
Total	-0.12	-0.47	0.22	Z = -0.707, P = 0.479	95

Figure S4: Sensitivity analysis of time to the first analgesic request comparing ESPB and QLB. CI, confidence interval.

Supplementary Figure 5

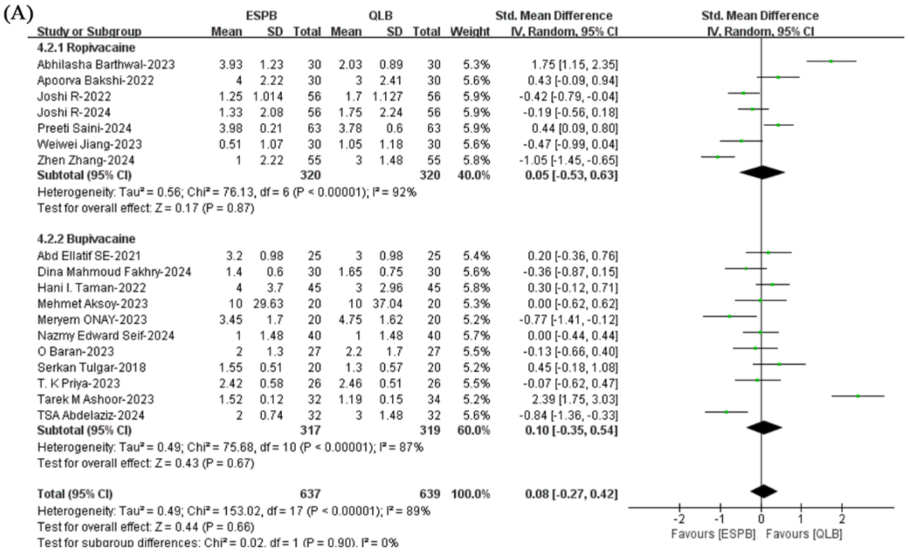

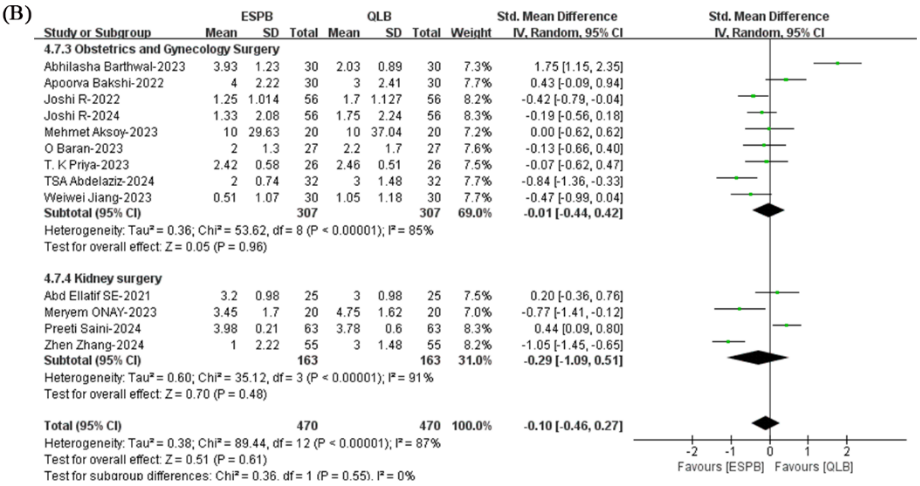

Figure S5: (A) Subgroup analysis for blocking drug of postoperative 6-h resting pain scores comparing ESPB and QLB. **(B)** Subgroup analysis for surgery type of postoperative 6-h resting pain scores comparing ESPB and QLB. ESPB, erector spinae plane block; QLB, quadratus lumborum block; SD, standard deviation; IV, inverse variance method; CI, confidence interval.

Supplementary Figure 6

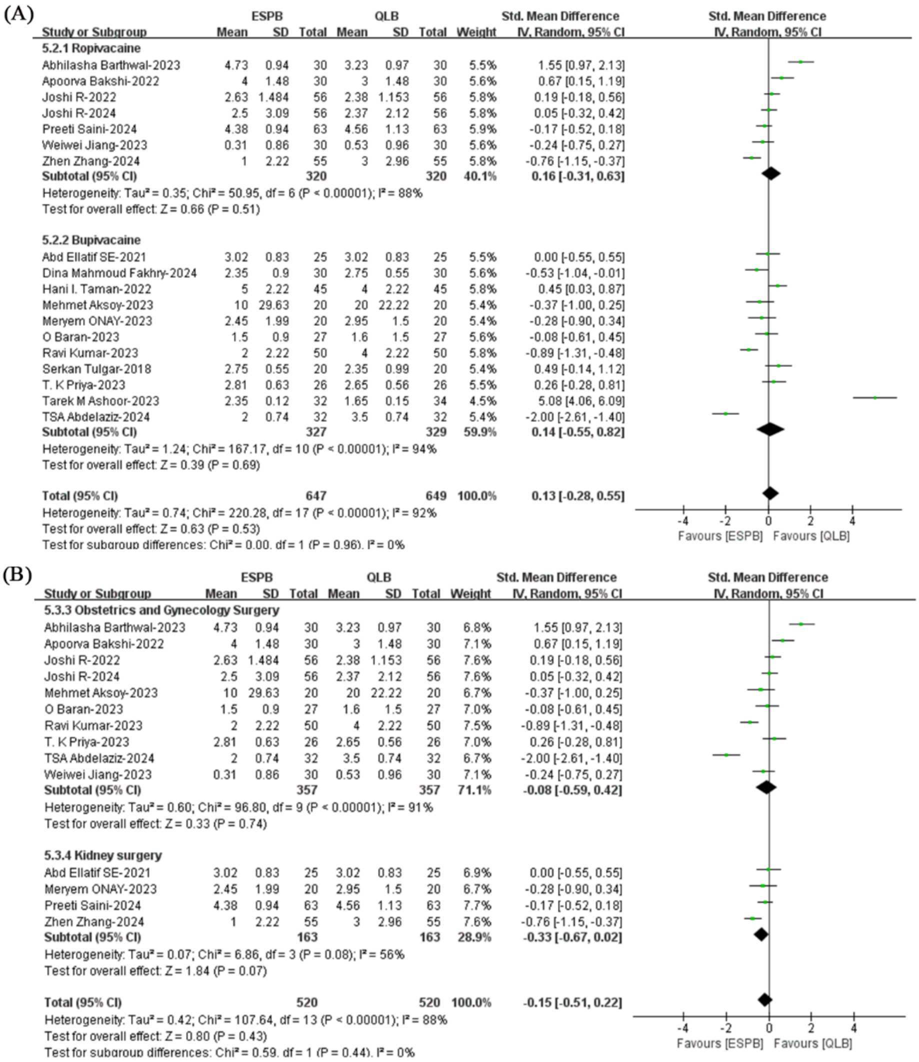

Figure S6: (A) Subgroup analysis for blocking drug of postoperative 12-h resting pain scores comparing ESPB and QLB. **(B)** Subgroup analysis for surgery type of postoperative 12-h resting pain scores comparing ESPB and QLB. ESPB, erector spinae plane block; QLB, quadratus lumborum block; SD, standard deviation; IV, inverse variance method; CI, confidence interval.

Supplementary Figure 7

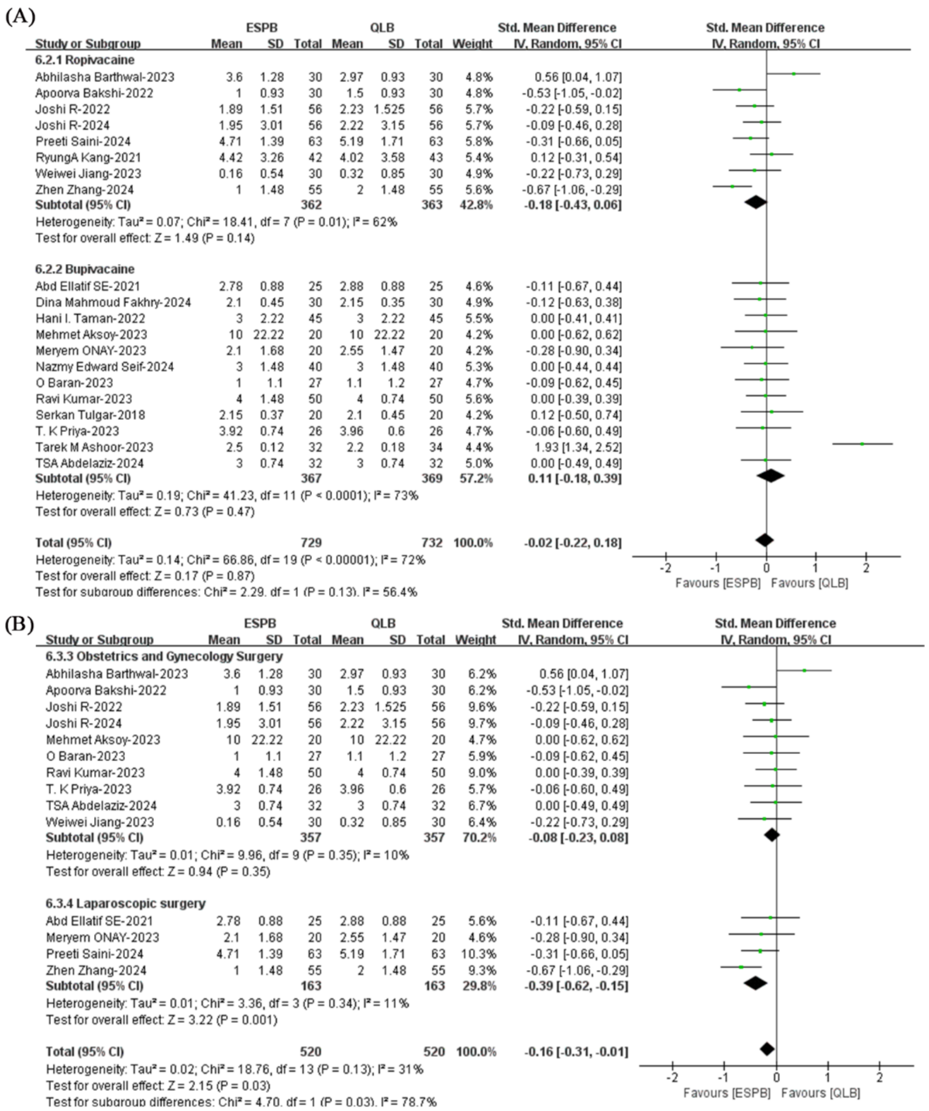

Figure S7: (A) Subgroup analysis for blocking drug of postoperative 24-h resting pain scores comparing ESPB and QLB. **(B)** Subgroup analysis for surgery type of postoperative 24-h resting pain scores comparing ESPB and QLB. ESPB, erector spinae plane block; QLB, quadratus lumborum block; SD, standard deviation; IV, inverse variance method; CI, confidence interval.

Supplementary Figure 8

Study	Effect Size	95% CI		P value	I^2(%)
(-)Abd Ellatif SE-2021	0.07	-0.29	0.43	Z = 0.383, P = 0.702	90
(-)Abhilasha Barthwal-2023	-0.02	-0.34	0.30	Z = -0.115, P = 0.908	87
(-)Apoorva Bakshi-2022	0.06	-0.30	0.42	Z = 0.311, P = 0.756	89
(-)Dina Mahmoud Fakhry-2024	0.10	-0.26	0.47	Z = 0.562, P = 0.574	89
(-)Hani I. Taman-2022	0.07	-0.30	0.43	Z = 0.346, P = 0.729	89
(-)Joshi R-2022	0.11	-0.26	0.48	Z = 0.583, P = 0.560	89
(-)Joshi R-2024	0.10	-0.28	0.47	Z = 0.503, P = 0.615	89
(-)Mehmet Aksoy-2023	0.08	-0.28	0.44	Z = 0.446, P = 0.656	90
(-)Meryem ONAY-2023	0.12	-0.23	0.48	Z = 0.684, P = 0.494	89
(-)Nazmy Edward Seif-2024	0.08	-0.29	0.45	Z = 0.442, P = 0.658	90
(-)O Baran-2023	0.09	-0.27	0.45	Z = 0.486, P = 0.627	90
(-)Preeti Saini-2024	0.06	-0.31	0.42	Z = 0.295, P = 0.768	89
(-)Serkan Tulgar-2018	0.06	-0.30	0.42	Z = 0.311, P = 0.756	89
(-)T. K Priya-2023	0.09	-0.28	0.45	Z = 0.468, P = 0.640	90
(-)Tarek M Ashoor-2023	-0.05	-0.34	0.24	Z = -0.358, P = 0.721	84
(-)TSA Abdelaziz-2024	0.13	-0.22	0.48	Z = 0.732, P = 0.464	89
(-)Weiwei Jiang-2023	0.11	-0.25	0.47	Z = 0.599, P = 0.549	89
(-)Zhen Zhang-2024	0.15	-0.19	0.48	Z = 0.856, P = 0.392	87
Total	0.08	-0.27	0.42	Z = 0.440, P = 0.660	89

Figure S8: Sensitivity analysis of postoperative 6-h resting pain scores comparing ESPB and QLB. CI, confidence interval.

Supplementary Figure 9

Study	Effect Size	95% CI		P value	I^2(%)
(-)Abd Ellatif SE-2021	0.14	-0.30	0.58	Z = 0.642, P = 0.521	93
(-)Abhilasha Barthwal-2023	0.05	-0.36	0.46	Z = 0.226, P = 0.821	92
(-)Apoorva Bakshi-2022	0.10	-0.33	0.54	Z = 0.467, P = 0.641	92
(-)Dina Mahmoud Fakhry-2024	0.18	-0.26	0.61	Z = 0.783, P = 0.433	93
(-)Hani I. Taman-2022	0.12	-0.33	0.56	Z = 0.521, P = 0.603	93
(-)Joshi R-2022	0.14	-0.32	0.59	Z = 0.587, P = 0.557	93
(-)Joshi R-2024	0.14	-0.31	0.60	Z = 0.624, P = 0.533	93
(-)Mehmet Aksoy-2023	0.17	-0.27	0.60	Z = 0.738, P = 0.460	93
(-)Meryem ONAY-2023	0.16	-0.28	0.60	Z = 0.714, P = 0.475	93
(-)O Baran-2023	0.15	-0.29	0.59	Z = 0.662, P = 0.508	93
(-)Preeti Saini-2024	0.16	-0.30	0.61	Z = 0.682, P = 0.495	93
(-)Ravi Kumar-2023	0.20	-0.23	0.63	Z = 0.898, P = 0.369	92
(-)Serkan Tulgar-2018	0.12	-0.32	0.55	Z = 0.518, P = 0.604	93
(-)T. K Priya-2023	0.13	-0.31	0.57	Z = 0.573, P = 0.567	93
(-)Tarek M Ashoor-2023	-0.10	-0.42	0.22	Z = -0.608, P = 0.543	87
(-)TSA Abdelaziz-2024	0.25	-0.15	0.64	Z = 1.227, P = 0.220	91
(-)Weiwei Jiang-2023	0.16	-0.28	0.60	Z = 0.704, P = 0.481	93
(-)Zhen Zhang-2024	0.19	-0.25	0.63	Z = 0.856, P = 0.392	92
Total	0.13	-0.28	0.55	Z = 0.629, P = 0.530	92

Figure S9: Sensitivity analysis of postoperative 12-h resting pain scores comparing ESPB and QLB. CI, confidence interval.

Supplementary Figure 10

Study	Effect Size	95% CI		P value	I^2(%)
(-)Abd Ellatif SE-2021	-0.01	-0.22	0.20	Z = 0.110, P = 0.912	73
(-)Abhilasha Barthwal-2023	-0.05	-0.25	0.15	Z = 0.460, P = 0.645	71
(-)Apoorva Bakshi-2022	0.01	-0.19	0.21	Z = 0.087, P = 0.931	72
(-)Dina Mahmoud Fakhry-2024	-0.01	-0.22	0.20	Z = 0.101, P = 0.920	73
(-)Hani I. Taman-2022	-0.02	-0.23	0.19	Z = 0.157, P = 0.875	73
(-)Joshi R-2022	0.00	-0.21	0.21	Z = 0.032, P = 0.974	73
(-)Joshi R-2024	-0.01	-0.22	0.20	Z = 0.106, P = 0.916	73
(-)Mehmet Aksoy-2023	-0.02	-0.22	0.19	Z = 0.162, P = 0.871	73
(-)Meryem ONAY-2023	-0.01	-0.21	0.20	Z = 0.047, P = 0.963	73
(-)Nazmy Edward Seif-2024	-0.02	-0.23	0.19	Z = 0.158, P = 0.874	73
(-)O Baran-2023	-0.01	-0.22	0.20	Z = 0.121, P = 0.904	73
(-)Preeti Saini-2024	0.00	-0.21	0.21	Z = 0.016, P = 0.987	72
(-)Ravi Kumar-2023	-0.02	-0.23	0.19	Z = 0.156, P = 0.876	73
(-)RyungA Kang-2021	-0.02	-0.23	0.19	Z = 0.221, P = 0.825	73
(-)Serkan Tulgar-2018	-0.02	-0.23	0.18	Z = 0.214, P = 0.831	73
(-)T. K Priya-2023	-0.01	-0.22	0.19	Z = 0.134, P = 0.893	73
(-)Tarek M Ashoor-2023	-0.12	-0.24	0.00	Z = 1.986, P = 0.047	18
(-)TSA Abdelaziz-2024	-0.02	-0.23	0.19	Z = 0.160, P = 0.873	73
(-)Weiwei Jiang-2023	-0.01	-0.21	0.20	Z = 0.054, P = 0.957	73
(-)Zhen Zhang-2024	0.02	-0.17	0.22	Z = 0.209, P = 0.835	68
Total	-0.02	-0.22	0.18	Z = 0.168, P = 0.867	72

Figure S10: Sensitivity analysis of postoperative 24-h resting pain scores comparing ESPB and QLB. CI, confidence interval.

Supplementary Figure 11

Study	Effect Size	95% CI		P value	I^2(%)
(-)Abd Ellatif SE-2021	-1.29	-2.37	-0.22	Z = 2.356, P = 0.018	97
(-)Dina Mahmoud Fakhry-2024	-1.47	-2.76	-0.19	Z = 2.255, P = 0.024	98
(-)Hakan Aygun-2020	-1.31	-2.49	-0.14	Z = 2.198, P = 0.028	98
(-)Hani I. Taman-2022	-1.76	-2.97	-0.55	Z = 2.841, P = 0.004	98
(-)Nazmy Edward Seif-2024	-1.71	-3.01	-0.42	Z = 2.589, P = 0.010	98
(-)O Baran-2023	-1.60	-2.83	-0.38	Z = 2.567, P = 0.010	98
(-)Preeti Saini-2024	-1.82	-3.01	-0.63	Z = 3.008, P = 0.003	98
(-)RyungA Kang-2021	-1.70	-3.02	-0.39	Z = 2.536, P = 0.011	98
(-)Tarek M Ashoor-2023	-1.06	-2.10	-0.01	Z = 1.985, P = 0.047	97
(-)TSA Abdelaziz-2024	-1.73	-2.93	-0.52	Z = 2.803, P = 0.005	98
Total	-1.55	-2.68	-0.41	Z = 2.673, P = 0.008	98

Figure S11: (A) Sensitivity analysis of block performance time comparing erector spinae plane block and quadratus lumborum block. CI, confidence interval.

Supplementary Figure 12

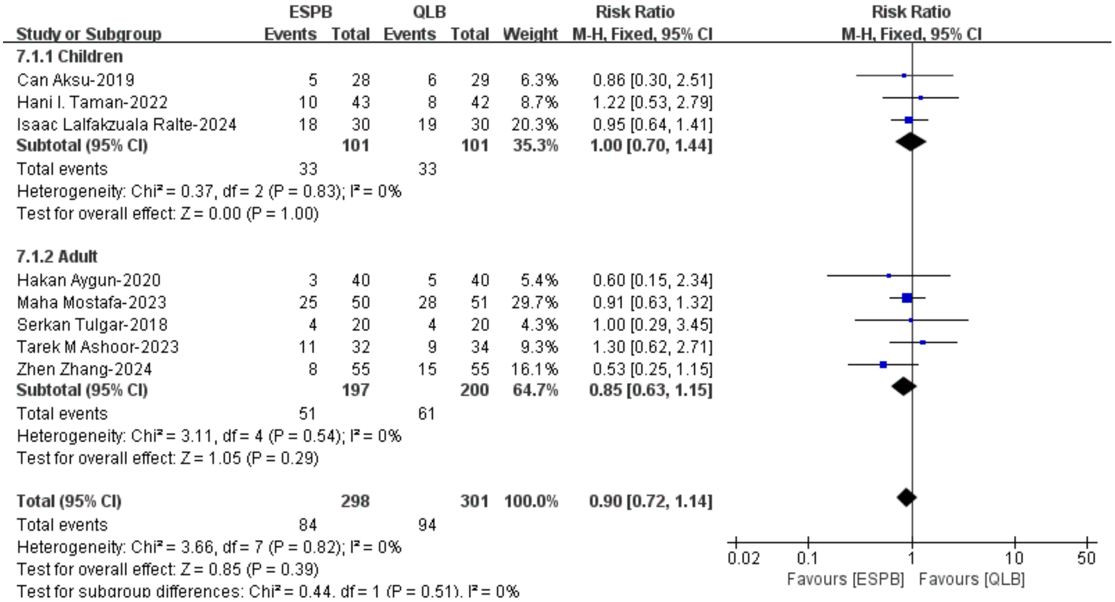

Figure S12: Subgroup analysis for age of postoperative rescue analgesia rate comparing ESPB and QLB. ESPB, erector spinae plane block; QLB, quadratus lumborum block; H-M, mantel-haenszel method; CI, confidence interval.

Supplementary Figure 13

Study	Effect Size	95% CI		P value	I^2(%)
(-)Can Aksu-2019	0.91	0.72	1.15	Z = 0.808, P = 0.419	0
(-)Hakan Aygun-2020	0.92	0.73	1.16	Z = 0.683, P = 0.495	0
(-)Hani I. Taman-2022	0.88	0.69	1.11	Z = 1.097, P = 0.273	0
(-)Isaac Lalfakzuala Ralte-2024	0.89	0.68	1.17	Z = 0.810, P = 0.418	0
(-)Maha Mostafa-2023	0.90	0.68	1.20	Z = 0.701, P = 0.483	0
(-)Serkan Tulgar-2018	0.90	0.71	1.14	Z = 0.878, P = 0.380	0
(-)Tarek M Ashoor-2023	0.86	0.68	1.10	Z = 1.179, P = 0.238	0
(-)Zhen Zhang-2024	0.98	0.77	1.24	Z = 0.199, P = 0.842	0
Total	0.91	0.72	1.14	Z = 0.735, P = 0.463	0

Figure S13: Sensitivity analysis of postoperative rescue analgesia rate comparing ESPB and QLB. CI, confidence interval

Supplementary Figure 14

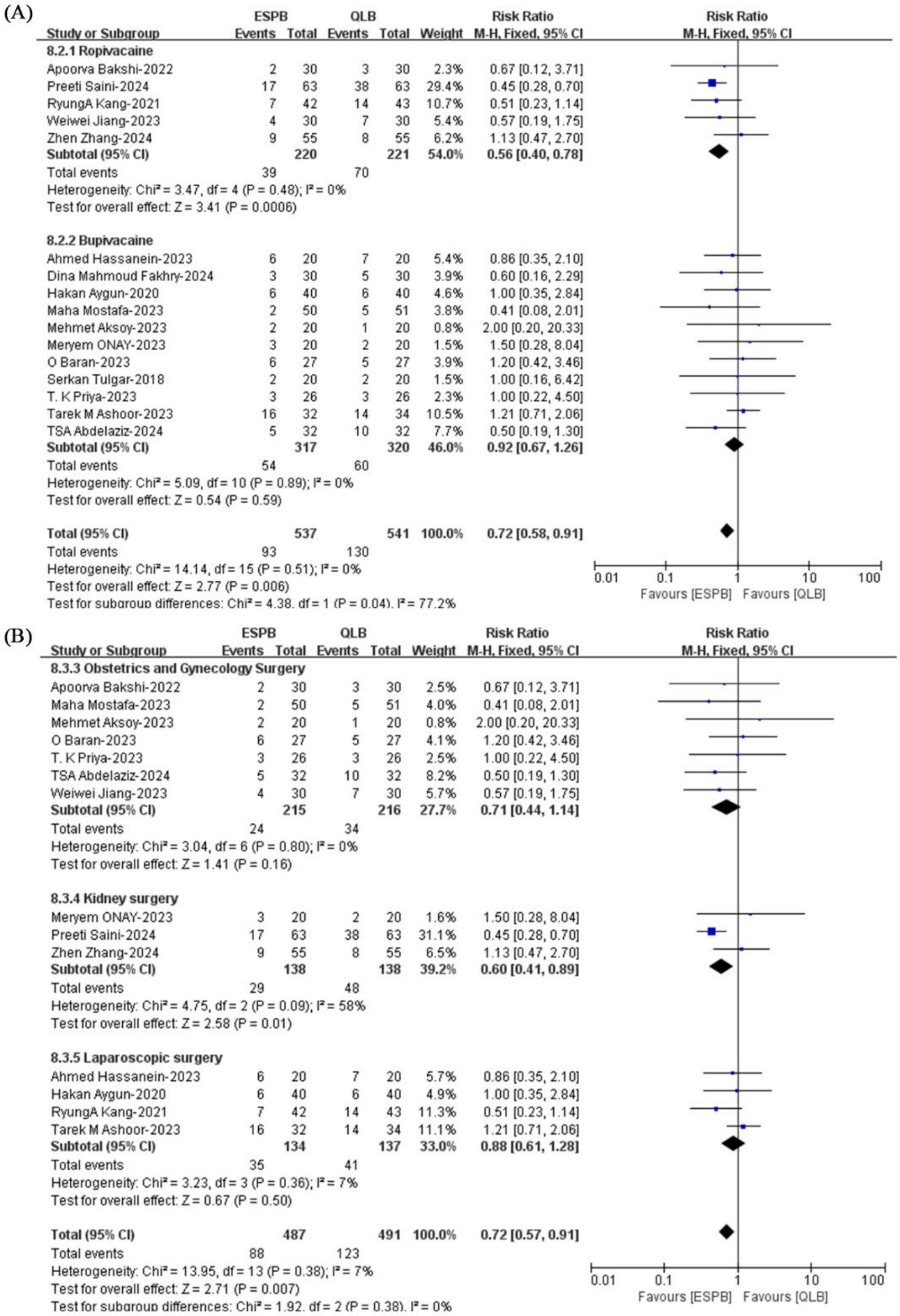

Figure S14: (A) Subgroup analysis for blocking drug of incidence of postoperative nausea and vomiting comparing ESPB and QLB. **(B)** Subgroup analysis for surgery type of incidence of postoperative nausea and vomiting comparing ESPB and QLB.

ESPB, erector spinae plane block; QLB, quadratus lumborum block; H-M, mantel-haenszel method; CI, confidence interval.

Supplementary Figure 15

Study	Effect Size	95% CI		P value	I^2(%)
(-)Ahmed Hassanein-2023	0.72	0.57	0.91	Z = -2.772, P = 0.006	0
(-)Hakan Aygun-2020	0.71	0.56	0.90	Z = -2.864, P = 0.004	0
(-)RyungA Kang-2021	0.75	0.59	0.95	Z = -2.374, P = 0.018	0
(-)Tarek M Ashoor-2023	0.67	0.52	0.86	Z = -3.154, P = 0.002	0
(-)Dina Mahmoud Fakhry-2024	0.73	0.58	0.92	Z = -2.678, P = 0.007	0
(-)Serkan Tulgar-2018	0.72	0.57	0.91	Z = -2.805, P = 0.005	0
(-)Apoorva Bakshi-2022	0.73	0.58	0.91	Z = -2.736, P = 0.006	1
(-)Maha Mostafa-2023	0.74	0.58	0.93	Z = -2.601, P = 0.009	0
(-)Mehmet Aksoy-2023	0.71	0.57	0.90	Z = -2.876, P = 0.004	0
(-)T. K Priya-2023	0.72	0.57	0.90	Z = -2.820, P = 0.005	0
(-)Preeti Saini-2024	0.84	0.64	1.10	Z = -1.293, P = 0.196	0
(-)Meryem ONAY-2023	0.71	0.57	0.90	Z = -2.890, P = 0.004	0
(-)Zhen Zhang-2024	0.70	0.55	0.88	Z = -2.983, P = 0.003	0
(-)O Baran-2023	0.70	0.56	0.89	Z = -2.930, P = 0.003	0
(-)TSA Abdelaziz-2024	0.74	0.59	0.94	Z = -2.480, P = 0.013	0
(-)Weiwei Jiang-2023	0.73	0.58	0.93	Z = -2.617, P = 0.009	0
Total	0.72	0.58	0.91	Z = -2.649, P = 0.008	0

Figure S15: Sensitivity analysis of incidence of postoperative nausea and vomiting comparing ESPB and QLB. CI, confidence interval.

Supplementary Figure 16

Study	Effect Size	95% CI		P value	I^2(%)
(-)Tarek M Ashoor-2023	0.66	0.31	1.39	Z = -1.100, P = 0.271	2
(-)Dina Mahmoud Fakhry-2024	0.63	0.25	1.61	Z = -0.961, P = 0.337	6
(-)T. K Priya-2023	0.49	0.22	1.10	Z = -1.721, P = 0.085	0
(-)Zhen Zhang-2024	0.74	0.36	1.52	Z = -0.813, P = 0.416	0
Total	0.63	0.32	1.25	Z = -1.090, P = 0.276	0

Figure S16: Sensitivity analysis of incidence of postoperative hypotension comparing ESPB and QLB. CI, confidence interval.

Supplementary Figure 17

Study	Effect Size	95% CI		P value	I^2(%)
(-)Abhilasha Barthwal-2023	1.03	0.92	1.15	Z = 0.471, P = 0.638	81
(-)Apoorva Bakshi-2022	0.98	0.84	1.13	Z = 0.311, P = 0.756	85
(-)Dina Mahmoud Fakhry-2024	0.91	0.78	1.07	Z = 1.165, P = 0.244	88
(-)Joshi R-2024	0.97	0.80	1.18	Z = 0.298, P = 0.766	85
(-)Maha Mostafa-2023	0.97	0.79	1.18	Z = 0.333, P = 0.739	85
(-)RyungA Kang-2021	0.99	0.87	1.12	Z = 0.224, P = 0.823	85
Total	0.98	0.87	1.10	Z = 0.422, P = 0.673	83

Figure S17: Sensitivity analysis of postoperative satisfaction rate comparing ESPB and QLB. CI, confidence interval.

Supplementary Figure 18

Study	Effect Size	95% CI		P value	I^2(%)
(-)Can Aksu-2019	-0.17	-0.38	0.03	Z = -1.677, P = 0.094	38
(-)Hani I. Taman-2022	-0.06	-0.27	0.15	Z = -0.572, P = 0.567	51
(-)Tarek M Ashoor-2023	-0.03	-0.24	0.17	Z = -0.314, P = 0.754	9
(-)Joshi R-2022	-0.16	-0.38	0.07	Z = -1.377, P = 0.169	56
(-)Joshi R-2024	-0.16	-0.38	0.07	Z = -1.377, P = 0.169	56
Total	-0.11	-0.30	0.08	Z = -0.918, P = 0.358	46

Figure S18: Sensitivity analysis of postoperative satisfaction comparing ESPB and QLB. CI, confidence interval.

Supplementary Figure 19

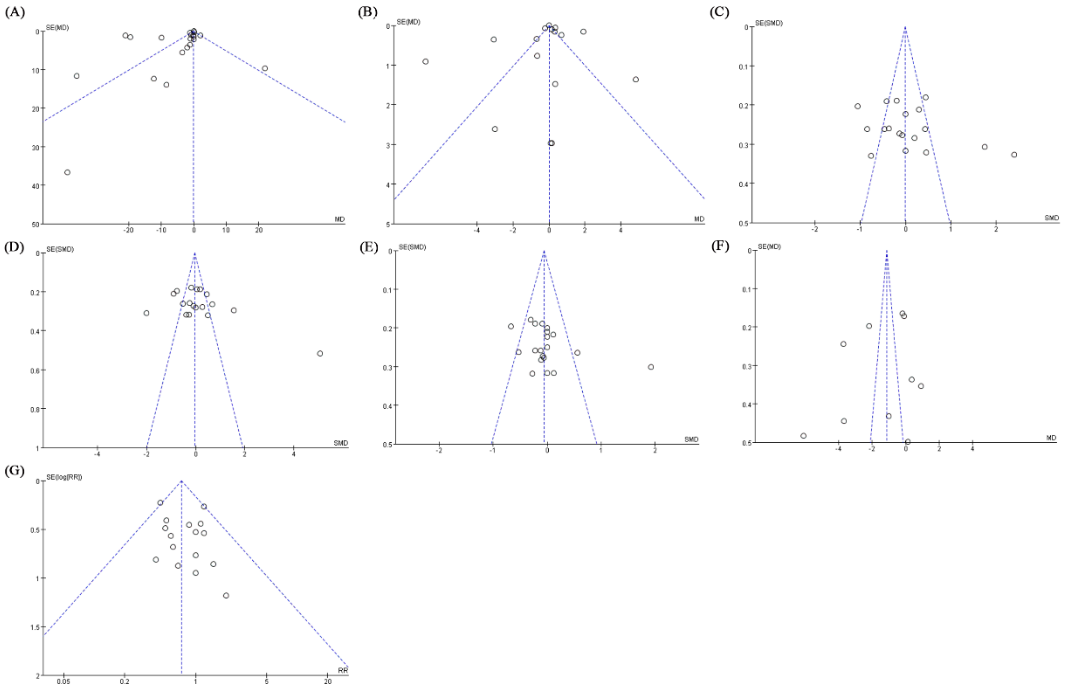

Figure S19: Funnel plots of publication bias of **(A)** postoperative analgesic consumption over 24 hours, **(B)** time to the first analgesic request, **(C)** postoperative resting pain scores at 6 hours, **(D)** 12 hours and **(E)** 24 hours, **(F)** block performance time, and **(G)** incidence of postoperative nausea and vomiting. SE, standard error; SMD, standardized mean difference; MD, mean difference; RR, risk ratio.
